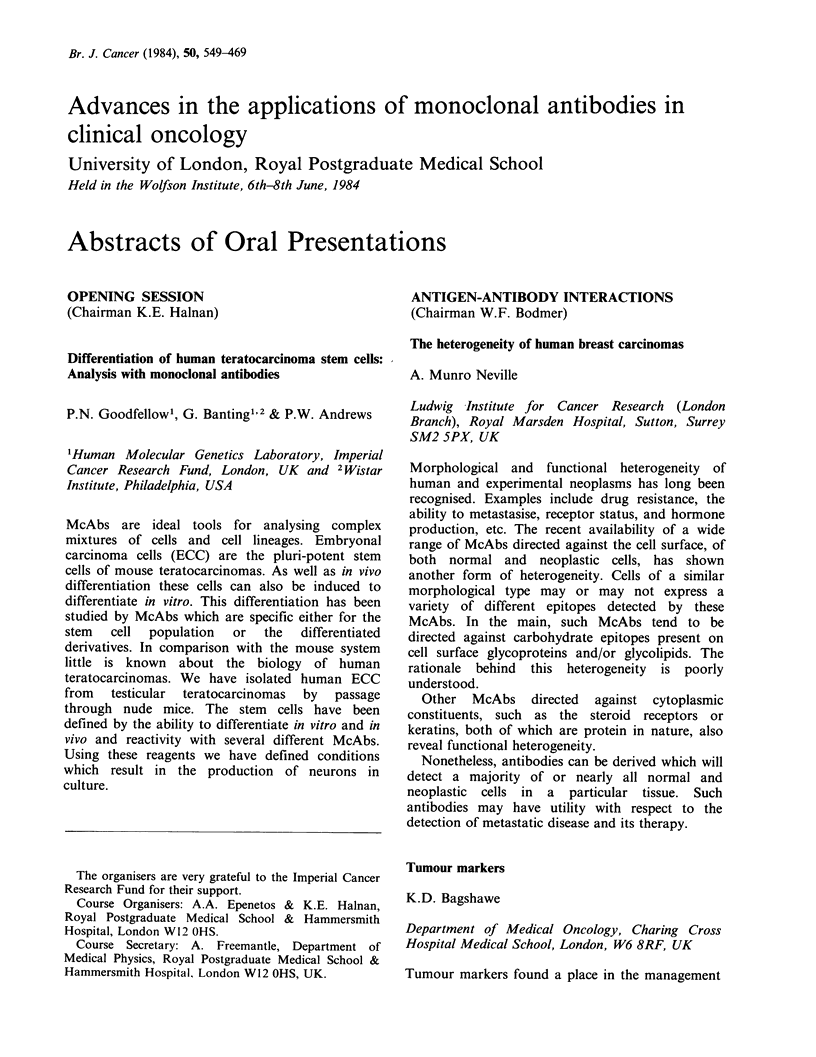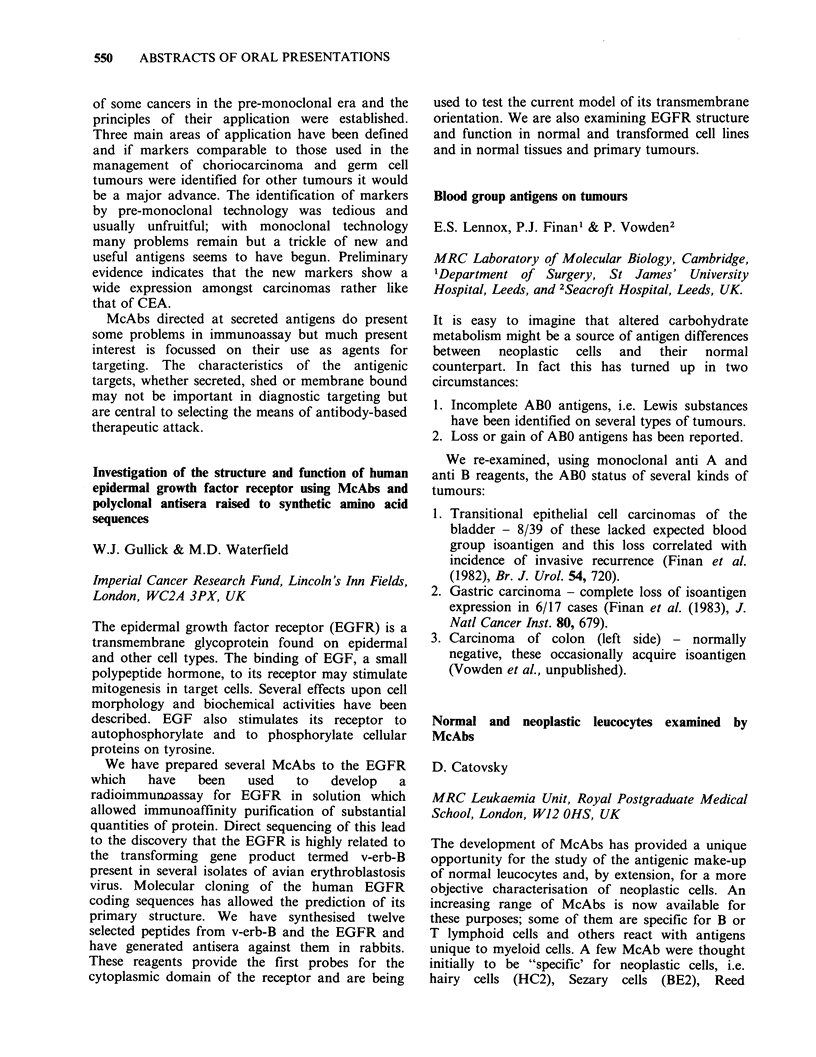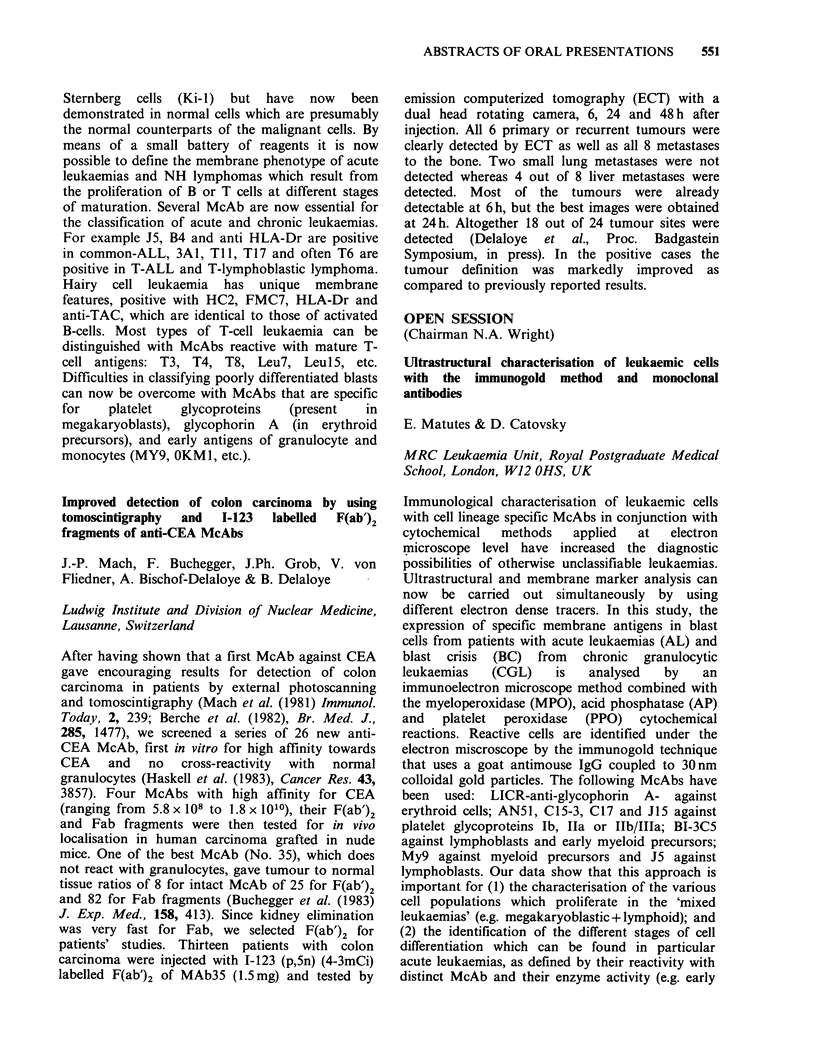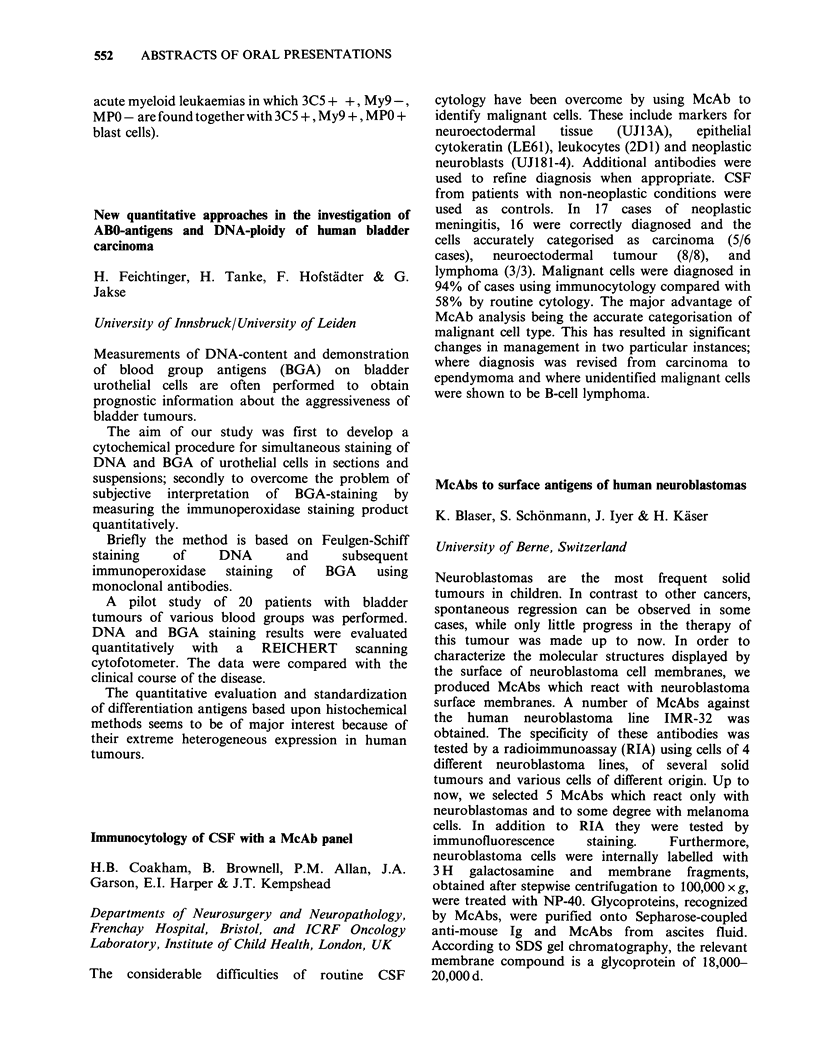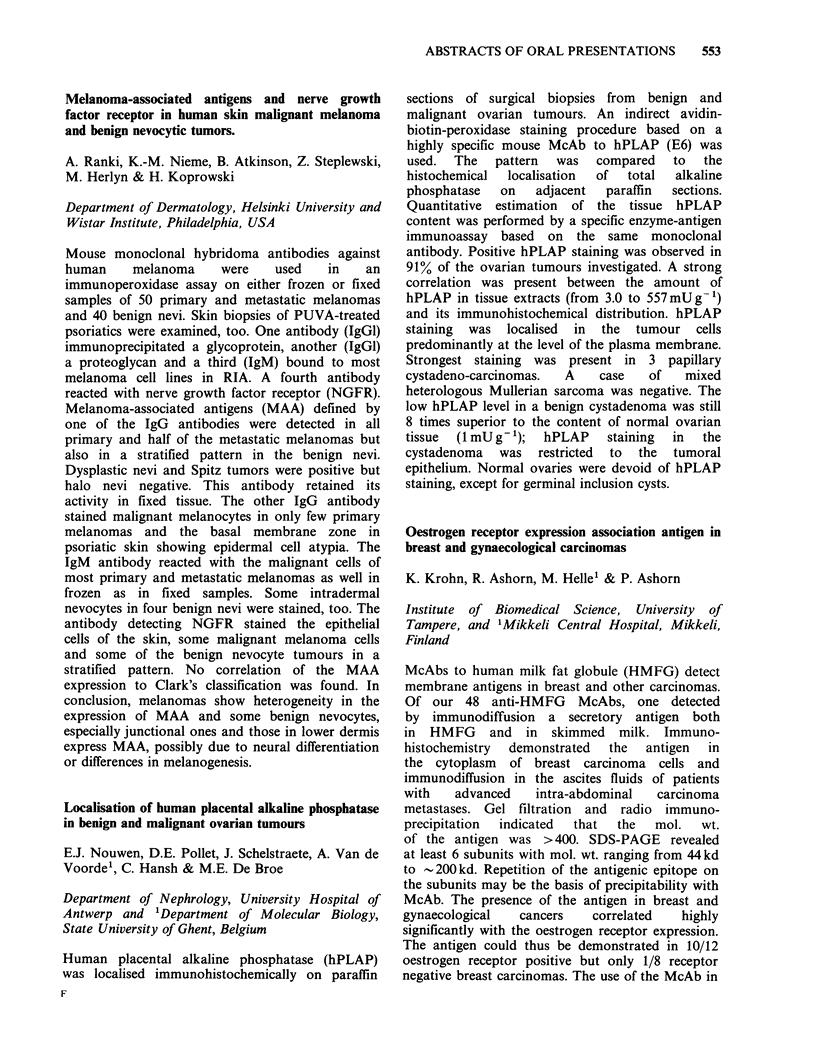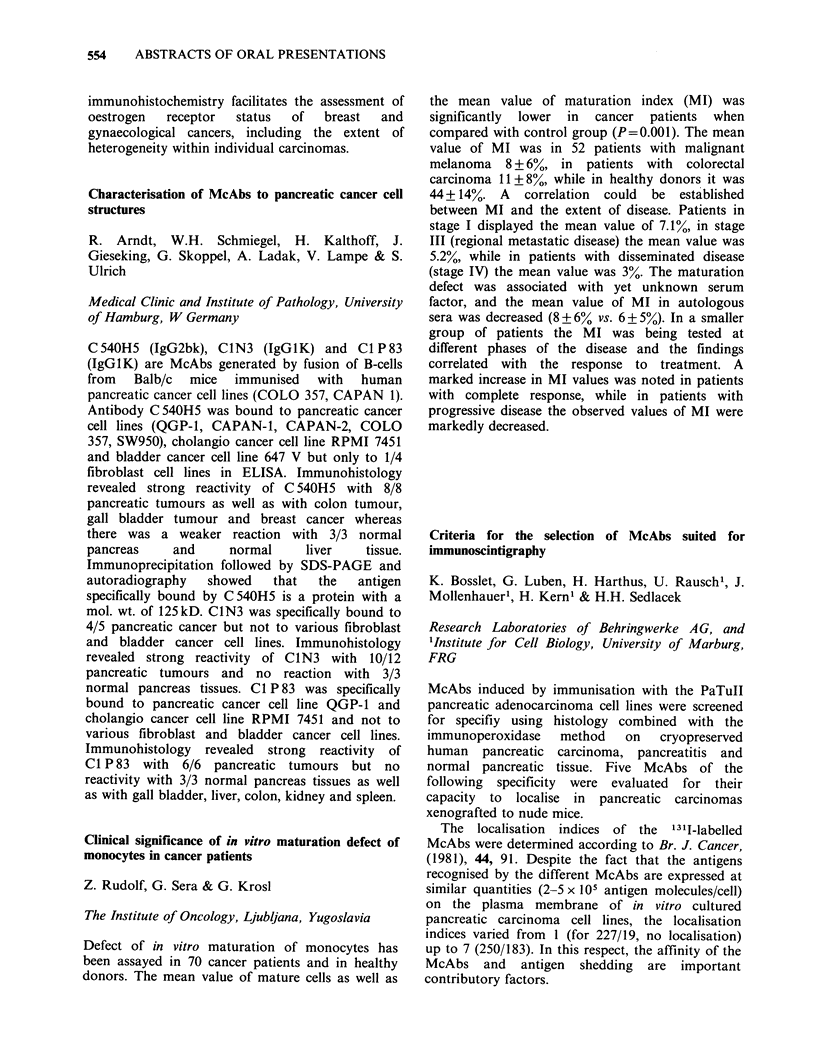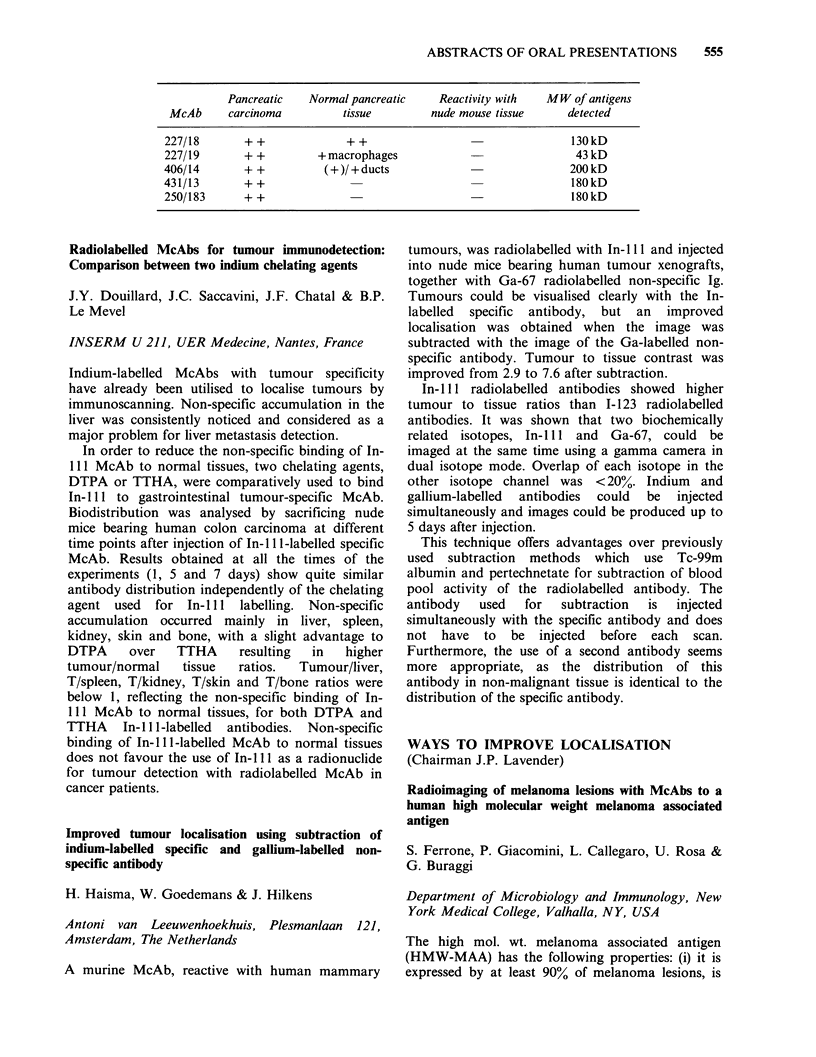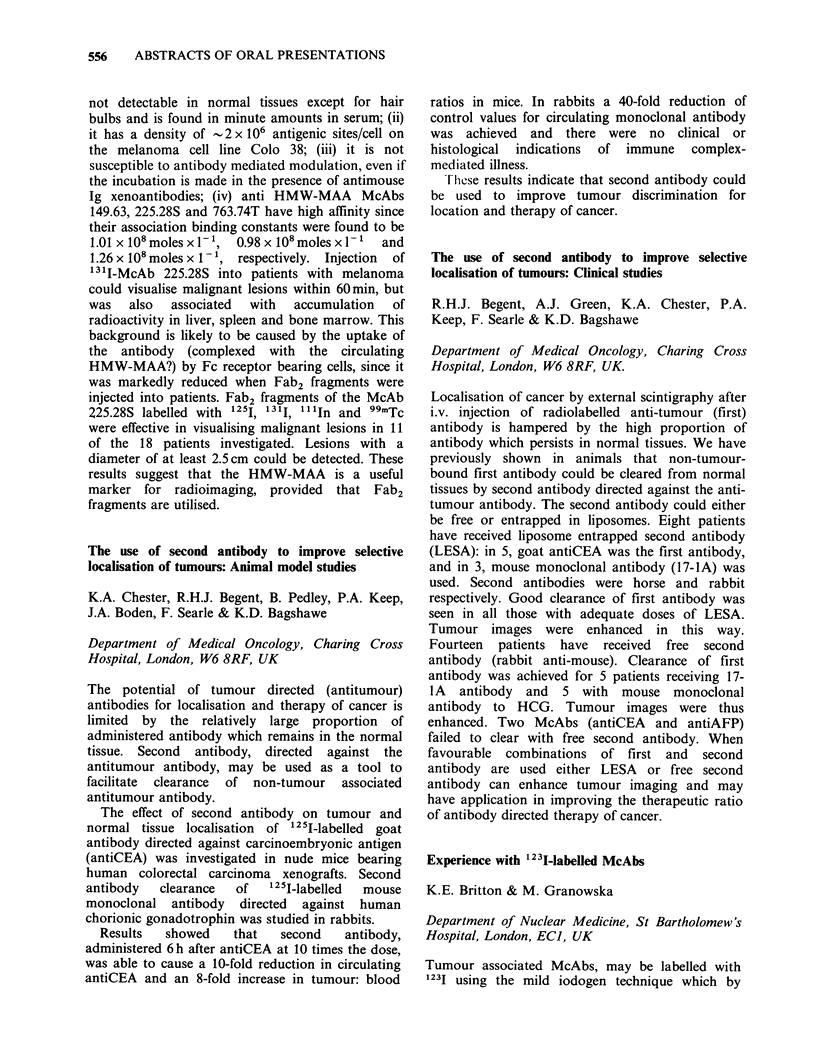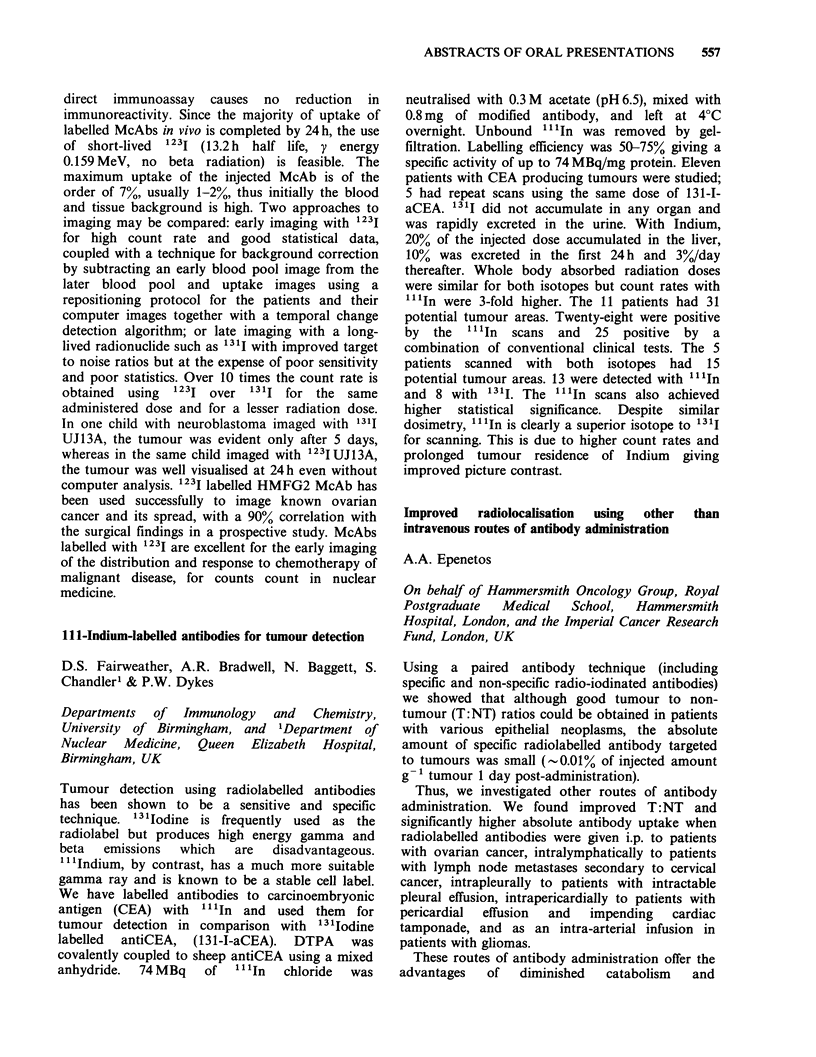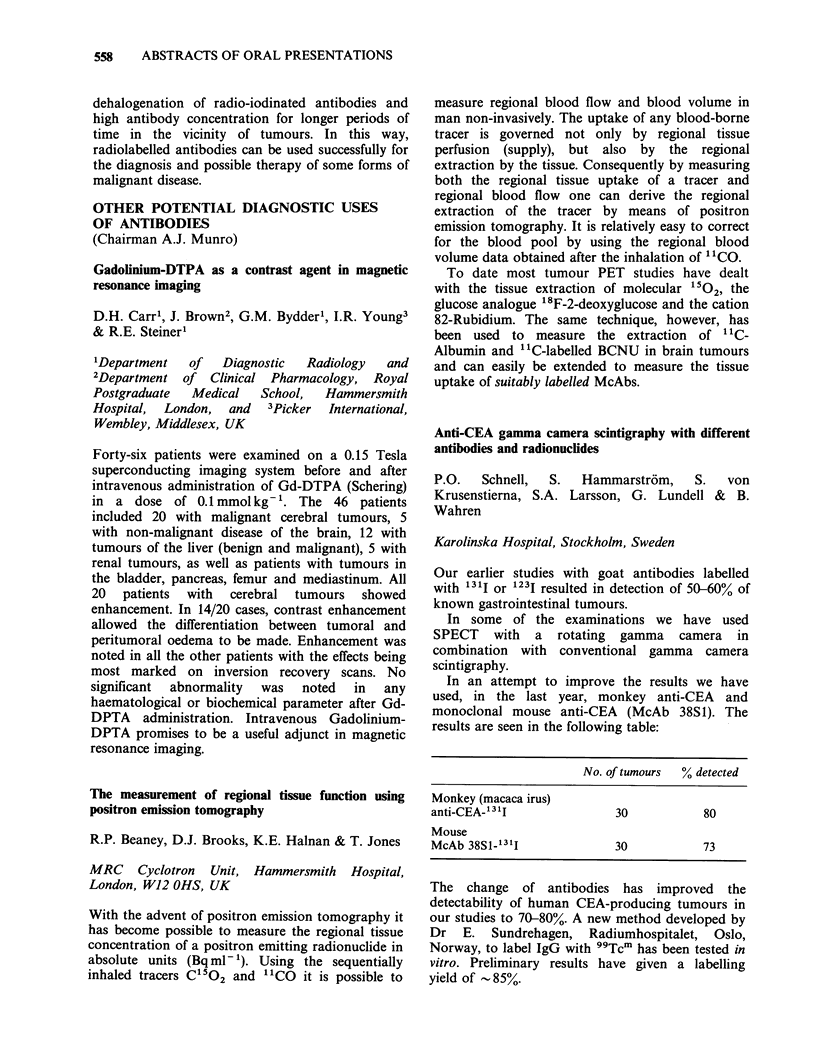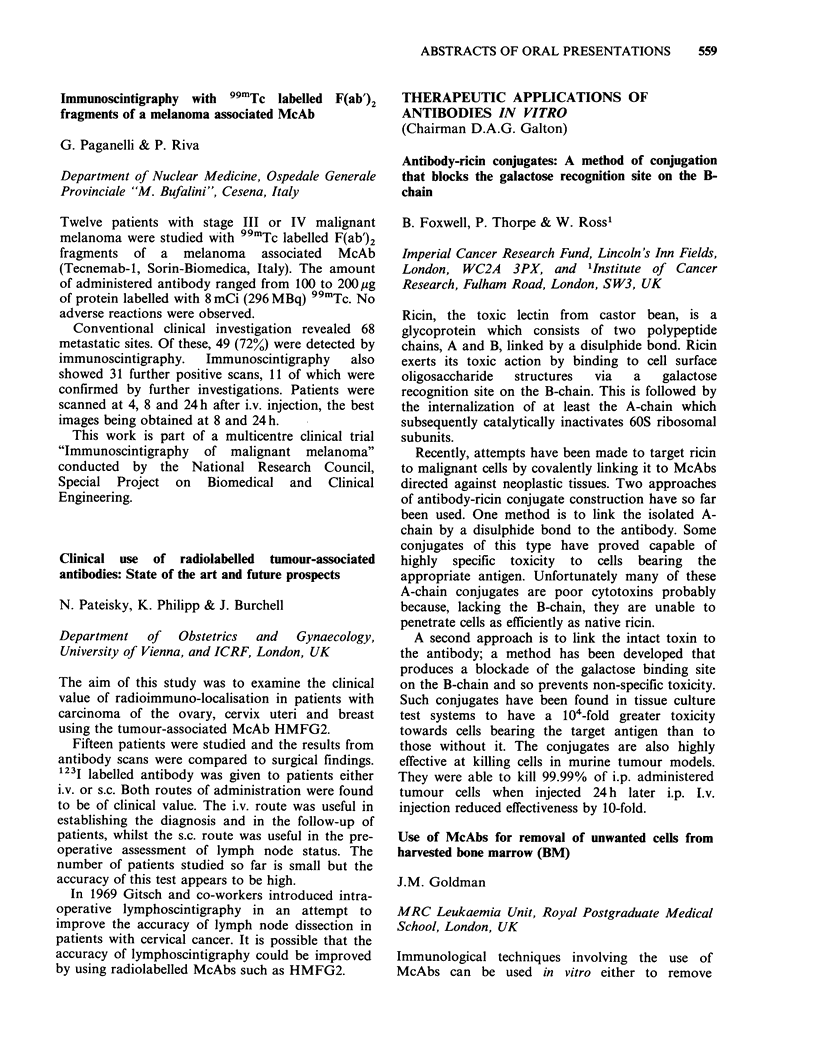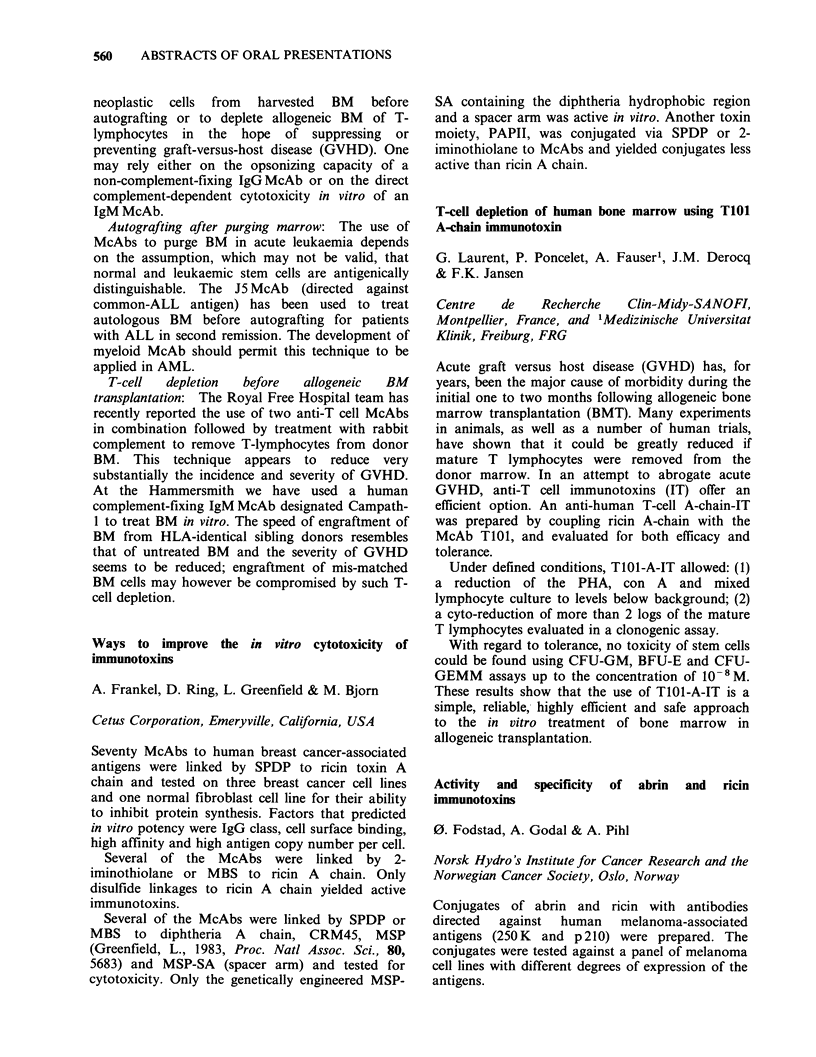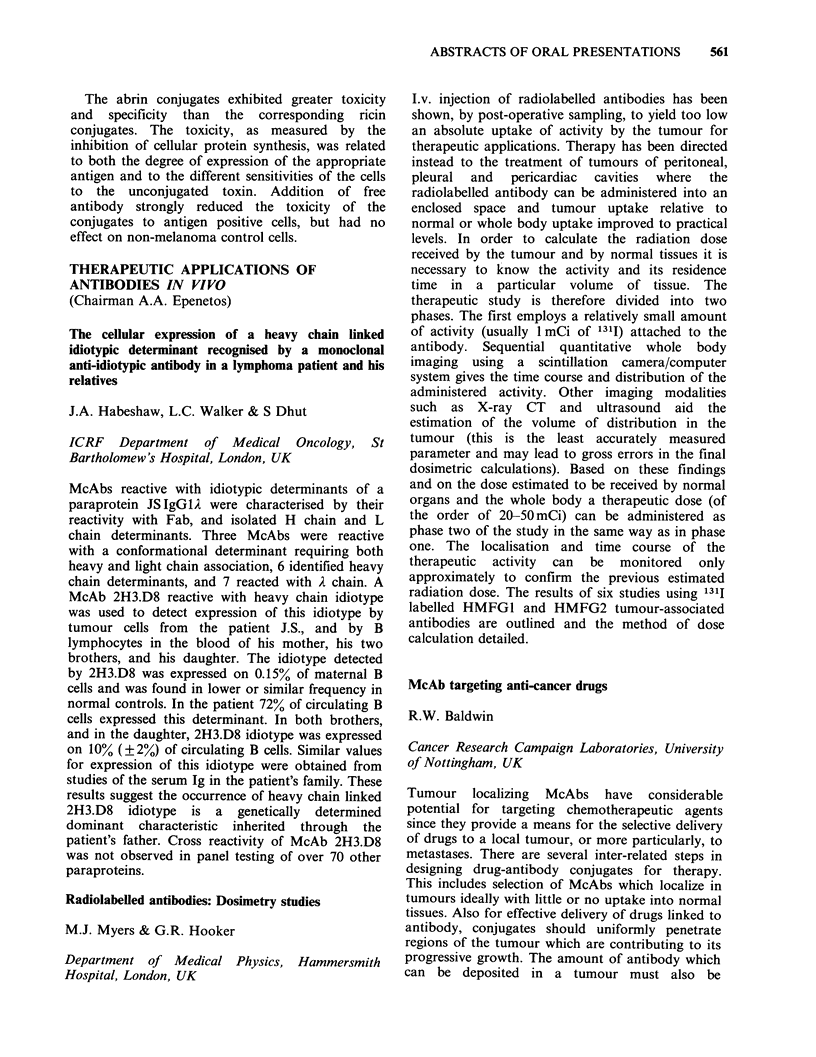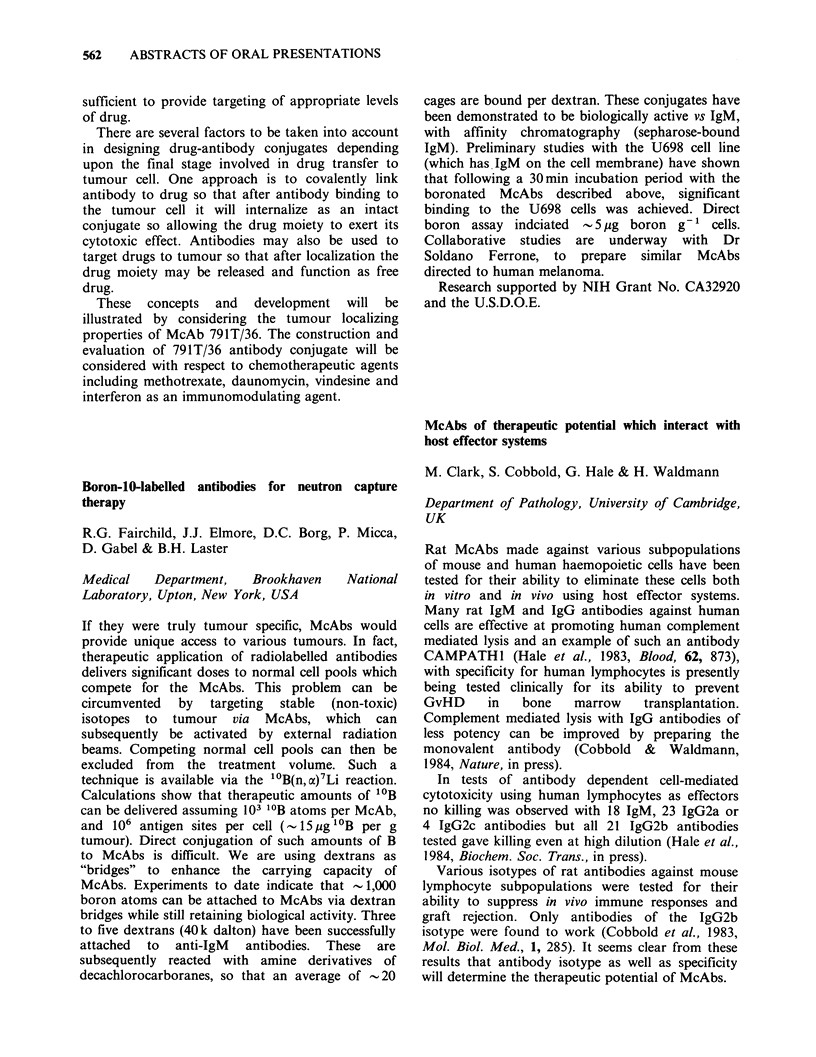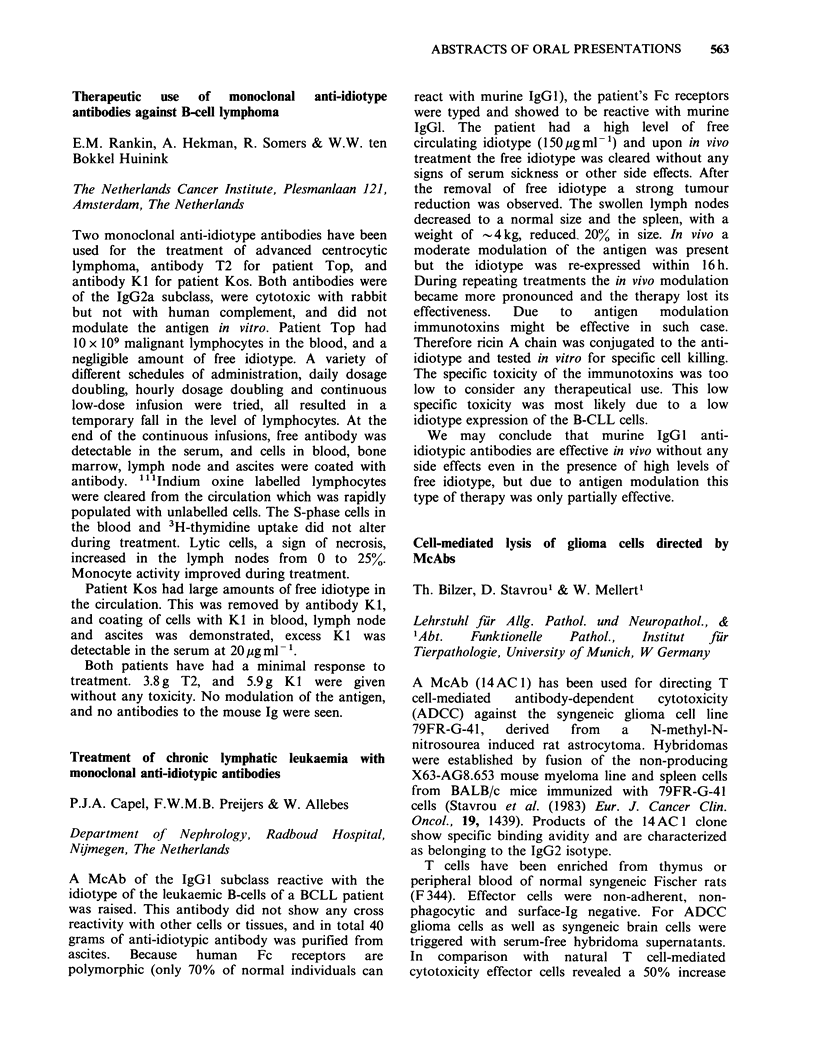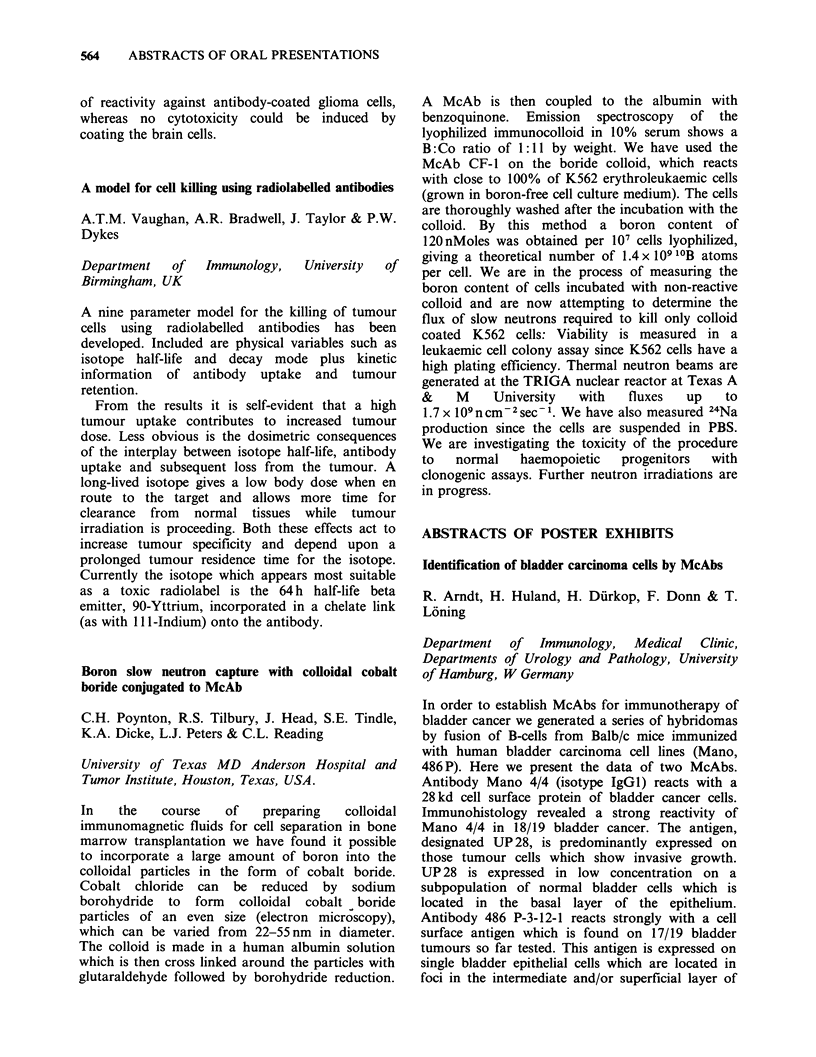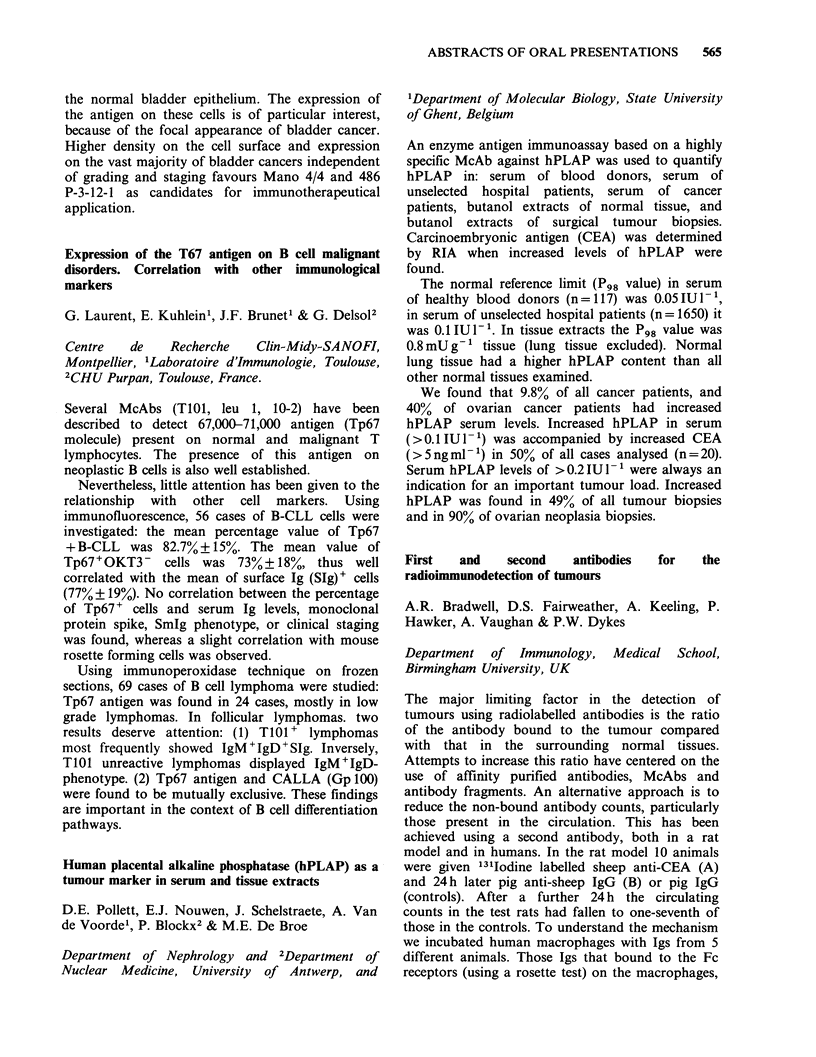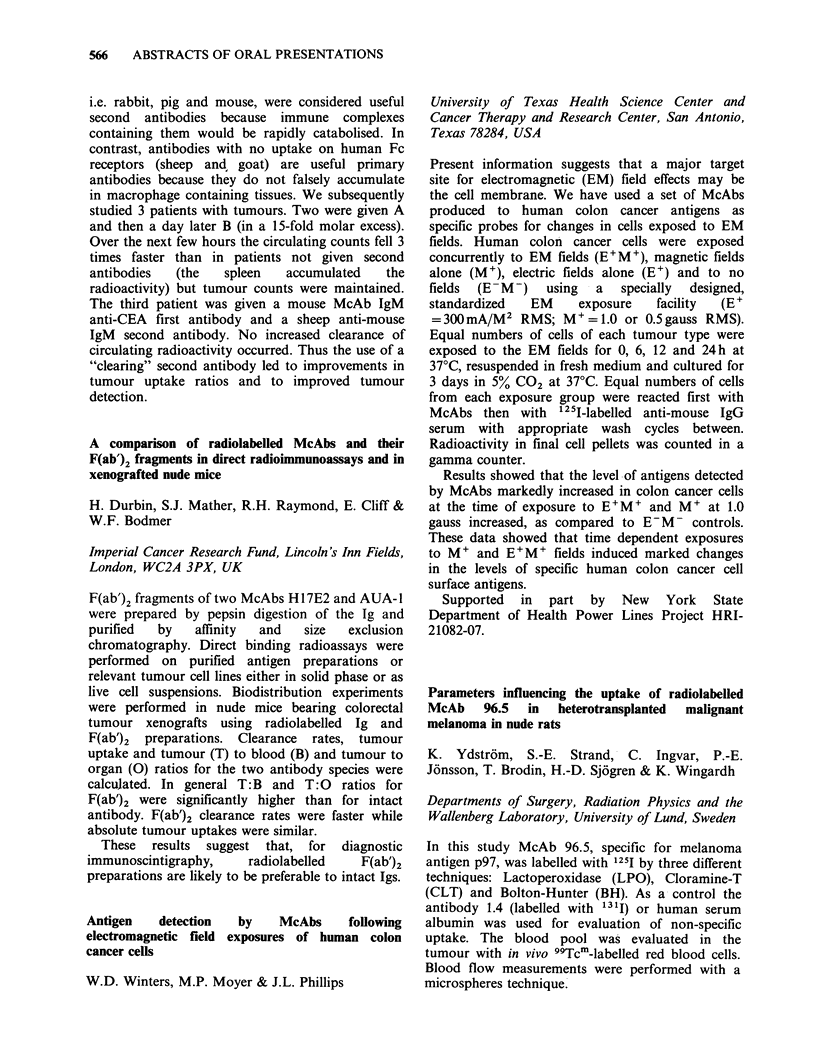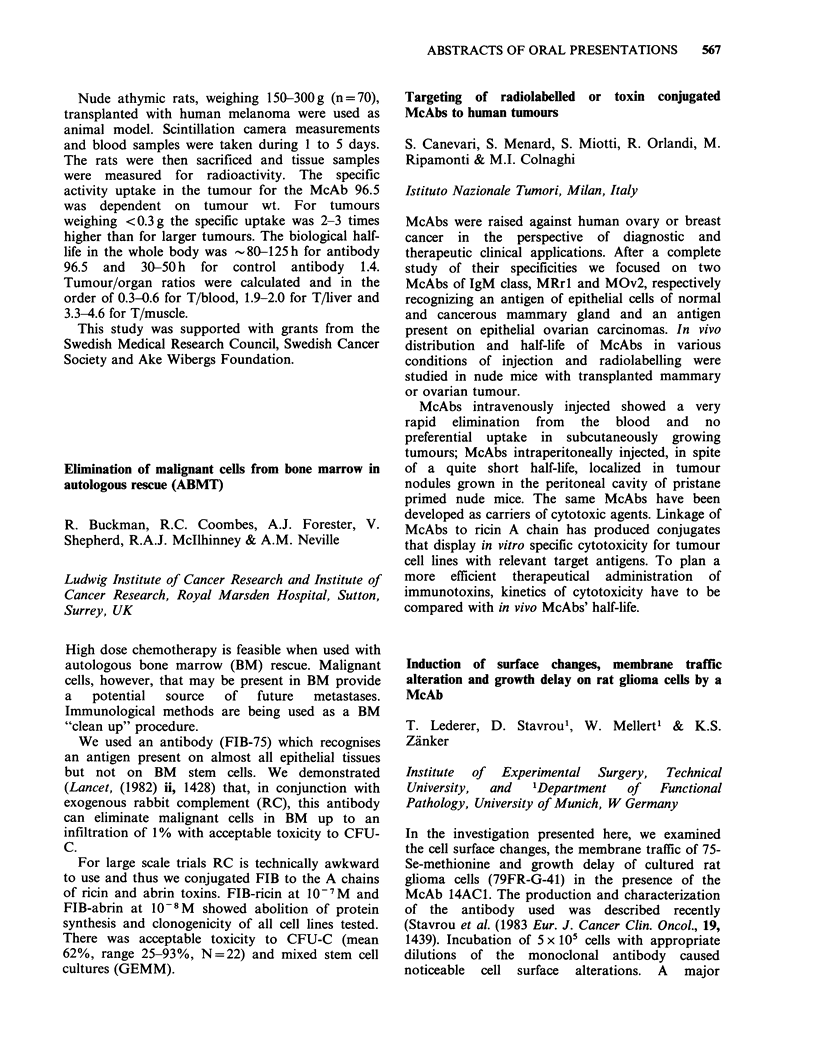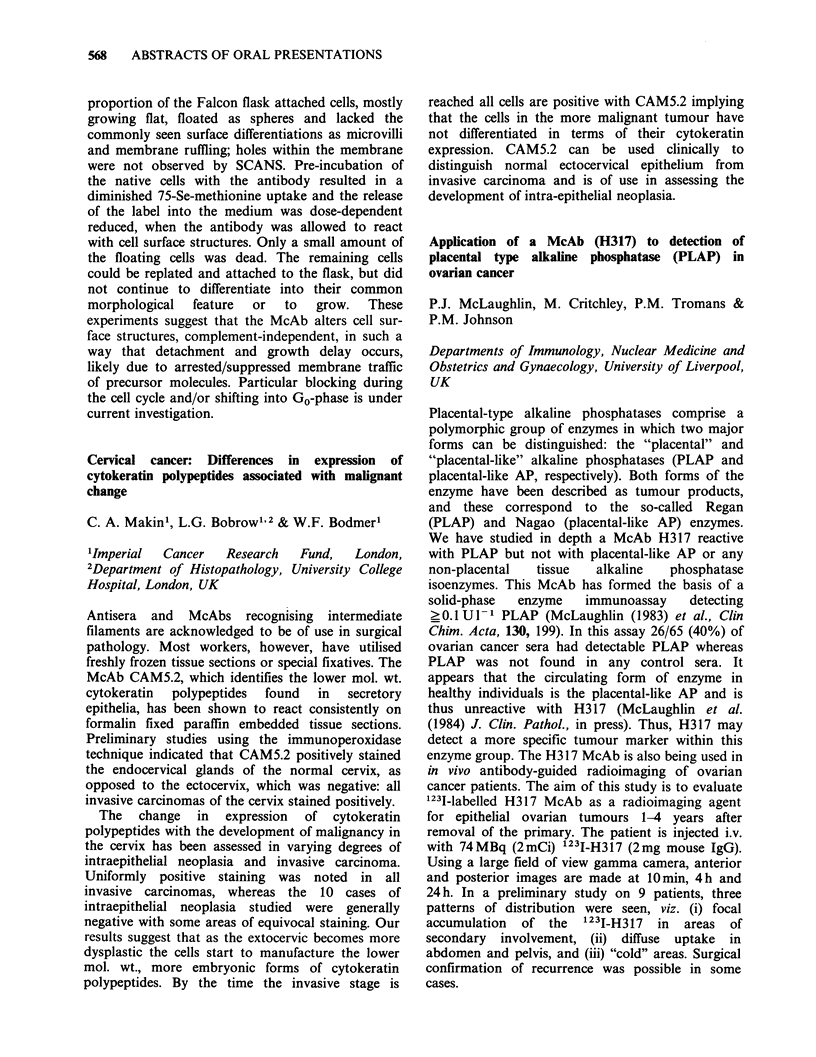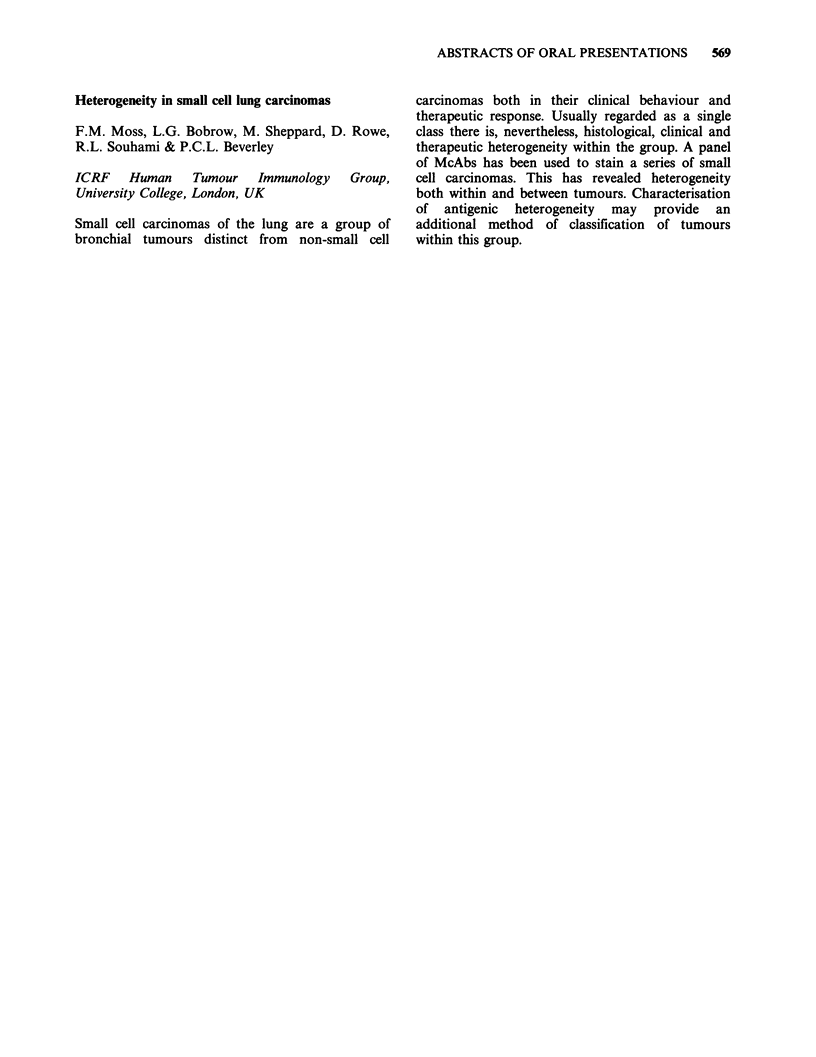# Advances in the applications of monoclonal antibodies in clinical oncology. Abstracts.

**DOI:** 10.1038/bjc.1984.215

**Published:** 1984-10

**Authors:** 


Br. J. Cancer (1984), 50, 549–469

### Advances in the applications of monoclonal antibodies in clinical oncology

### University of London, Royal Postgraduate Medical School

*Held in the Wolfson Institute, 6th–8th June, 1984*

### Abstracts of Oral Presentations

### OPENING SESSION
(Chairman K.E. Halnan)

### Differentiation of human teratocarcinoma stem cells: Analysis with monoclonal antibodies

P.N. Goodfellow[1], G. Banting[1,2] & P.W. Andrews

[1]*Human Molecular Genetics Laboratory, Imperial Cancer Research Fund, London, UK and* [2]*Wistar Institute, Philadelphia, USA*

McAbs are ideal tools for analysing complex mixtures of cells and cell lineages. Embryonal carcinoma cells (ECC) are the pluri-potent stem cells of mouse teratocarcinomas. As well as *in vivo* differentiation these cells can also be induced to differentiate *in vitro*. This differentiation has been studied by McAbs which are specific either for the stem cell population or the differentiated derivatives. In comparison with the mouse system little is known about the biology of human teratocarcinomas. We have isolated human ECC from testicular teratocarcinomas by passage through nude mice. The stem cells have been defined by the ability to differentiate *in vitro* and *in vivo* and reactivity with several different McAbs. Using these reagents we have defined conditions which result in the production of neurons in culture.

---

The organisers are very grateful to the Imperial Cancer Research Fund for their support.

Course Organisers: A.A. Epenetos & K.E. Halnan, Royal Postgraduate Medical School & Hammersmith Hospital, London W12 0HS.

Course Secretary: A. Freemantle, Department of Medical Physics, Royal Postgraduate Medical School & Hammersmith Hospital, London W12 0HS, UK.

### ANTIGEN-ANTIBODY INTERACTIONS
(Chairman W.F. Bodmer)

### The heterogeneity of human breast carcinomas

A. Munro Neville

*Ludwig Institute for Cancer Research (London Branch), Royal Marsden Hospital, Sutton, Surrey SM2 5PX, UK*

Morphological and functional heterogeneity of human and experimental neoplasms has long been recognised. Examples include drug resistance, the ability to metastasise, receptor status, and hormone production, etc. The recent availability of a wide range of McAbs directed against the cell surface, of both normal and neoplastic cells, has shown another form of heterogeneity. Cells of a similar morphological type may or may not express a variety of different epitopes detected by these McAbs. In the main, such McAbs tend to be directed against carbohydrate epitopes present on cell surface glycoproteins and/or glycolipids. The rationale behind this heterogeneity is poorly understood.

Other McAbs directed against cytoplasmic constituents, such as the steroid receptors or keratins, both of which are protein in nature, also reveal functional heterogeneity.

Nonetheless, antibodies can be derived which will detect a majority of or nearly all normal and neoplastic cells in a particular tissue. Such antibodies may have utility with respect to the detection of metastatic disease and its therapy.

### Tumour markers

K.D. Bagshawe

*Department of Medical Oncology, Charing Cross Hospital Medical School, London, W6 8RF, UK*

Tumour markers found a place in the management of some cancers in the pre-monoclonal era and the principles of their application were established. Three main areas of application have been defined and if markers comparable to those used in the management of choriocarcinoma and germ cell tumours were identified for other tumours it would be a major advance. The identification of markers by pre-monoclonal technology was tedious and usually unfruitful; with monoclonal technology many problems remain but a trickle of new and useful antigens seems to have begun. Preliminary evidence indicates that the new markers show a wide expression amongst carcinomas rather like that of CEA.

McAbs directed at secreted antigens do present some problems in immunoassay but much present interest is focussed on their use as agents for targeting. The characteristics of the antigenic targets, whether secreted, shed or membrane bound may not be important in diagnostic targeting but are central to selecting the means of antibody-based therapeutic attack.

**Investigation of the structure and function of human epidermal growth factor receptor using McAbs and polyclonal antisera raised to synthetic amino acid sequences**

W.J. Gullick & M.D. Waterfield

*Imperial Cancer Research Fund, Lincoln's Inn Fields, London, WC2A 3PX, UK*

The epidermal growth factor receptor (EGFR) is a transmembrane glycoprotein found on epidermal and other cell types. The binding of EGF, a small polypeptide hormone, to its receptor may stimulate mitogenesis in target cells. Several effects upon cell morphology and biochemical activities have been described. EGF also stimulates its receptor to autophosphorylate and to phosphorylate cellular proteins on tyrosine.

We have prepared several McAbs to the EGFR which have been used to develop a radioimmunoassay for EGFR in solution which allowed immunoaffinity purification of substantial quantities of protein. Direct sequencing of this lead to the discovery that the EGFR is highly related to the transforming gene product termed v-erb-B present in several isolates of avian erythroblastosis virus. Molecular cloning of the human EGFR coding sequences has allowed the prediction of its primary structure. We have synthesised twelve selected peptides from v-erb-B and the EGFR and have generated antisera against them in rabbits. These reagents provide the first probes for the cytoplasmic domain of the receptor and are being used to test the current model of its transmembrane orientation. We are also examining EGFR structure and function in normal and transformed cell lines and in normal tissues and primary tumours.

**Blood group antigens on tumours**

E.S. Lennox, P.J. Finan[1] & P. Vowden[2]

*MRC Laboratory of Molecular Biology, Cambridge, [1]Department of Surgery, St James' University Hospital, Leeds, and [2]Seacroft Hospital, Leeds, UK.*

It is easy to imagine that altered carbohydrate metabolism might be a source of antigen differences between neoplastic cells and their normal counterpart. In fact this has turned up in two circumstances:

1. Incomplete AB0 antigens, i.e. Lewis substances have been identified on several types of tumours.
2. Loss or gain of AB0 antigens has been reported.

We re-examined, using monoclonal anti A and anti B reagents, the AB0 status of several kinds of tumours:

1. Transitional epithelial cell carcinomas of the bladder – 8/39 of these lacked expected blood group isoantigen and this loss correlated with incidence of invasive recurrence (Finan *et al.* (1982), *Br. J. Urol.* **54,** 720).
2. Gastric carcinoma – complete loss of isoantigen expression in 6/17 cases (Finan *et al.* (1983), *J. Natl Cancer Inst.* **80,** 679).
3. Carcinoma of colon (left side) – normally negative, these occasionally acquire isoantigen (Vowden *et al.*, unpublished).

**Normal and neoplastic leucocytes examined by McAbs**

D. Catovsky

*MRC Leukaemia Unit, Royal Postgraduate Medical School, London, W12 0HS, UK*

The development of McAbs has provided a unique opportunity for the study of the antigenic make-up of normal leucocytes and, by extension, for a more objective characterisation of neoplastic cells. An increasing range of McAbs is now available for these purposes; some of them are specific for B or T lymphoid cells and others react with antigens unique to myeloid cells. A few McAb were thought initially to be "specific' for neoplastic cells, i.e. hairy cells (HC2), Sezary cells (BE2), Reed

Sternberg cells (Ki-1) but have now been demonstrated in normal cells which are presumably the normal counterparts of the malignant cells. By means of a small battery of reagents it is now possible to define the membrane phenotype of acute leukaemias and NH lymphomas which result from the proliferation of B or T cells at different stages of maturation. Several McAb are now essential for the classification of acute and chronic leukaemias. For example J5, B4 and anti HLA-Dr are positive in common-ALL, 3A1, T11, T17 and often T6 are positive in T-ALL and T-lymphoblastic lymphoma. Hairy cell leukaemia has unique membrane features, positive with HC2, FMC7, HLA-Dr and anti-TAC, which are identical to those of activated B-cells. Most types of T-cell leukaemia can be distinguished with McAbs reactive with mature T-cell antigens: T3, T4, T8, Leu7, Leu15, etc. Difficulties in classifying poorly differentiated blasts can now be overcome with McAbs that are specific for platelet glycoproteins (present in megakaryoblasts), glycophorin A (in erythroid precursors), and early antigens of granulocyte and monocytes (MY9, 0KM1, etc.).

**Improved detection of colon carcinoma by using tomoscintigraphy and I-123 labelled $F(ab')_2$ fragments of anti-CEA McAbs**

J.-P. Mach, F. Buchegger, J.Ph. Grob, V. von Fliedner, A. Bischof-Delaloye & B. Delaloye

*Ludwig Institute and Division of Nuclear Medicine, Lausanne, Switzerland*

After having shown that a first McAb against CEA gave encouraging results for detection of colon carcinoma in patients by external photoscanning and tomoscintigraphy (Mach *et al.* (1981) *Immunol. Today*, **2,** 239; Berche *et al.* (1982), *Br. Med. J.*, **285,** 1477), we screened a series of 26 new anti-CEA McAb, first *in vitro* for high affinity towards CEA and no cross-reactivity with normal granulocytes (Haskell *et al.* (1983), *Cancer Res.* **43,** 3857). Four McAbs with high affinity for CEA (ranging from $5.8 \times 10^8$ to $1.8 \times 10^{10}$), their $F(ab')_2$ and Fab fragments were then tested for *in vivo* localisation in human carcinoma grafted in nude mice. One of the best McAb (No. 35), which does not react with granulocytes, gave tumour to normal tissue ratios of 8 for intact McAb of 25 for $F(ab')_2$ and 82 for Fab fragments (Buchegger *et al.* (1983) *J. Exp. Med.*, **158,** 413). Since kidney elimination was very fast for Fab, we selected $F(ab')_2$ for patients' studies. Thirteen patients with colon carcinoma were injected with I-123 (p,5n) (4-3mCi) labelled $F(ab')_2$ of MAb35 (1.5 mg) and tested by emission computerized tomography (ECT) with a dual head rotating camera, 6, 24 and 48 h after injection. All 6 primary or recurrent tumours were clearly detected by ECT as well as all 8 metastases to the bone. Two small lung metastases were not detected whereas 4 out of 8 liver metastases were detected. Most of the tumours were already detectable at 6 h, but the best images were obtained at 24 h. Altogether 18 out of 24 tumour sites were detected (Delaloye *et al.*, Proc. Badgastein Symposium, in press). In the positive cases the tumour definition was markedly improved as compared to previously reported results.

### OPEN SESSION

(Chairman N.A. Wright)

**Ultrastructural characterisation of leukaemic cells with the immunogold method and monoclonal antibodies**

E. Matutes & D. Catovsky

*MRC Leukaemia Unit, Royal Postgraduate Medical School, London, W12 0HS, UK*

Immunological characterisation of leukaemic cells with cell lineage specific McAbs in conjunction with cytochemical methods applied at electron microscope level have increased the diagnostic possibilities of otherwise unclassifiable leukaemias. Ultrastructural and membrane marker analysis can now be carried out simultaneously by using different electron dense tracers. In this study, the expression of specific membrane antigens in blast cells from patients with acute leukaemias (AL) and blast crisis (BC) from chronic granulocytic leukaemias (CGL) is analysed by an immunoelectron microscope method combined with the myeloperoxidase (MPO), acid phosphatase (AP) and platelet peroxidase (PPO) cytochemical reactions. Reactive cells are identified under the electron miscroscope by the immunogold technique that uses a goat antimouse IgG coupled to 30 nm colloidal gold particles. The following McAbs have been used: LICR-anti-glycophorin A- against erythroid cells; AN51, C15-3, C17 and J15 against platelet glycoproteins Ib, IIa or IIb/IIIa; BI-3C5 against lymphoblasts and early myeloid precursors; My9 against myeloid precursors and J5 against lymphoblasts. Our data show that this approach is important for (1) the characterisation of the various cell populations which proliferate in the 'mixed leukaemias' (e.g. megakaryoblastic + lymphoid); and (2) the identification of the different stages of cell differentiation which can be found in particular acute leukaemias, as defined by their reactivity with distinct McAb and their enzyme activity (e.g. early acute myeloid leukaemias in which 3C5+ +, My9−, MP0− are found together with 3C5+, My9+, MP0+ blast cells).

### New quantitative approaches in the investigation of AB0-antigens and DNA-ploidy of human bladder carcinoma

H. Feichtinger, H. Tanke, F. Hofstädter & G. Jakse

*University of Innsbruck/University of Leiden*

Measurements of DNA-content and demonstration of blood group antigens (BGA) on bladder urothelial cells are often performed to obtain prognostic information about the aggressiveness of bladder tumours.

The aim of our study was first to develop a cytochemical procedure for simultaneous staining of DNA and BGA of urothelial cells in sections and suspensions; secondly to overcome the problem of subjective interpretation of BGA-staining by measuring the immunoperoxidase staining product quantitatively.

Briefly the method is based on Feulgen-Schiff staining of DNA and subsequent immunoperoxidase staining of BGA using monoclonal antibodies.

A pilot study of 20 patients with bladder tumours of various blood groups was performed. DNA and BGA staining results were evaluated quantitatively with a REICHERT scanning cytofotometer. The data were compared with the clinical course of the disease.

The quantitative evaluation and standardization of differentiation antigens based upon histochemical methods seems to be of major interest because of their extreme heterogeneous expression in human tumours.

### Immunocytology of CSF with a McAb panel

H.B. Coakham, B. Brownell, P.M. Allan, J.A. Garson, E.I. Harper & J.T. Kempshead

*Departments of Neurosurgery and Neuropathology, Frenchay Hospital, Bristol, and ICRF Oncology Laboratory, Institute of Child Health, London, UK*

The considerable difficulties of routine CSF cytology have been overcome by using McAb to identify malignant cells. These include markers for neuroectodermal tissue (UJ13A), epithelial cytokeratin (LE61), leukocytes (2D1) and neoplastic neuroblasts (UJ181-4). Additional antibodies were used to refine diagnosis when appropriate. CSF from patients with non-neoplastic conditions were used as controls. In 17 cases of neoplastic meningitis, 16 were correctly diagnosed and the cells accurately categorised as carcinoma (5/6 cases), neuroectodermal tumour (8/8), and lymphoma (3/3). Malignant cells were diagnosed in 94% of cases using immunocytology compared with 58% by routine cytology. The major advantage of McAb analysis being the accurate categorisation of malignant cell type. This has resulted in significant changes in management in two particular instances; where diagnosis was revised from carcinoma to ependymoma and where unidentified malignant cells were shown to be B-cell lymphoma.

### McAbs to surface antigens of human neuroblastomas

K. Blaser, S. Schönmann, J. Iyer & H. Käser

*University of Berne, Switzerland*

Neuroblastomas are the most frequent solid tumours in children. In contrast to other cancers, spontaneous regression can be observed in some cases, while only little progress in the therapy of this tumour was made up to now. In order to characterize the molecular structures displayed by the surface of neuroblastoma cell membranes, we produced McAbs which react with neuroblastoma surface membranes. A number of McAbs against the human neuroblastoma line IMR-32 was obtained. The specificity of these antibodies was tested by a radioimmunoassay (RIA) using cells of 4 different neuroblastoma lines, of several solid tumours and various cells of different origin. Up to now, we selected 5 McAbs which react only with neuroblastomas and to some degree with melanoma cells. In addition to RIA they were tested by immunofluorescence staining. Furthermore, neuroblastoma cells were internally labelled with 3H galactosamine and membrane fragments, obtained after stepwise centrifugation to $100{,}000 \times g$, were treated with NP-40. Glycoproteins, recognized by McAbs, were purified onto Sepharose-coupled anti-mouse Ig and McAbs from ascites fluid. According to SDS gel chromatography, the relevant membrane compound is a glycoprotein of 18,000–20,000 d.

**Melanoma-associated antigens and nerve growth factor receptor in human skin malignant melanoma and benign nevocytic tumors.**

A. Ranki, K.-M. Nieme, B. Atkinson, Z. Steplewski, M. Herlyn & H. Koprowski

*Department of Dermatology, Helsinki University and Wistar Institute, Philadelphia, USA*

Mouse monoclonal hybridoma antibodies against human melanoma were used in an immunoperoxidase assay on either frozen or fixed samples of 50 primary and metastatic melanomas and 40 benign nevi. Skin biopsies of PUVA-treated psoriatics were examined, too. One antibody (IgGl) immunoprecipitated a glycoprotein, another (IgGl) a proteoglycan and a third (IgM) bound to most melanoma cell lines in RIA. A fourth antibody reacted with nerve growth factor receptor (NGFR). Melanoma-associated antigens (MAA) defined by one of the IgG antibodies were detected in all primary and half of the metastatic melanomas but also in a stratified pattern in the benign nevi. Dysplastic nevi and Spitz tumors were positive but halo nevi negative. This antibody retained its activity in fixed tissue. The other IgG antibody stained malignant melanocytes in only few primary melanomas and the basal membrane zone in psoriatic skin showing epidermal cell atypia. The IgM antibody reacted with the malignant cells of most primary and metastatic melanomas as well in frozen as in fixed samples. Some intradermal nevocytes in four benign nevi were stained, too. The antibody detecting NGFR stained the epithelial cells of the skin, some malignant melanoma cells and some of the benign nevocyte tumours in a stratified pattern. No correlation of the MAA expression to Clark's classification was found. In conclusion, melanomas show heterogeneity in the expression of MAA and some benign nevocytes, especially junctional ones and those in lower dermis express MAA, possibly due to neural differentiation or differences in melanogenesis.

**Localisation of human placental alkaline phosphatase in benign and malignant ovarian tumours**

E.J. Nouwen, D.E. Pollet, J. Schelstraete, A. Van de Voorde[1], C. Hansh & M.E. De Broe

*Department of Nephrology, University Hospital of Antwerp and [1]Department of Molecular Biology, State University of Ghent, Belgium*

Human placental alkaline phosphatase (hPLAP) was localised immunohistochemically on paraffin sections of surgical biopsies from benign and malignant ovarian tumours. An indirect avidin-biotin-peroxidase staining procedure based on a highly specific mouse McAb to hPLAP (E6) was used. The pattern was compared to the histochemical localisation of total alkaline phosphatase on adjacent paraffin sections. Quantitative estimation of the tissue hPLAP content was performed by a specific enzyme-antigen immunoassay based on the same monoclonal antibody. Positive hPLAP staining was observed in 91% of the ovarian tumours investigated. A strong correlation was present between the amount of hPLAP in tissue extracts (from 3.0 to 557 $mU\,g^{-1}$) and its immunohistochemical distribution. hPLAP staining was localised in the tumour cells predominantly at the level of the plasma membrane. Strongest staining was present in 3 papillary cystadeno-carcinomas. A case of mixed heterologous Mullerian sarcoma was negative. The low hPLAP level in a benign cystadenoma was still 8 times superior to the content of normal ovarian tissue (1 $mU\,g^{-1}$); hPLAP staining in the cystadenoma was restricted to the tumoral epithelium. Normal ovaries were devoid of hPLAP staining, except for germinal inclusion cysts.

**Oestrogen receptor expression association antigen in breast and gynaecological carcinomas**

K. Krohn, R. Ashorn, M. Helle[1] & P. Ashorn

*Institute of Biomedical Science, University of Tampere, and [1]Mikkeli Central Hospital, Mikkeli, Finland*

McAbs to human milk fat globule (HMFG) detect membrane antigens in breast and other carcinomas. Of our 48 anti-HMFG McAbs, one detected by immunodiffusion a secretory antigen both in HMFG and in skimmed milk. Immuno-histochemistry demonstrated the antigen in the cytoplasm of breast carcinoma cells and immunodiffusion in the ascites fluids of patients with advanced intra-abdominal carcinoma metastases. Gel filtration and radio immuno-precipitation indicated that the mol. wt. of the antigen was >400. SDS-PAGE revealed at least 6 subunits with mol. wt. ranging from 44 kd to ~200 kd. Repetition of the antigenic epitope on the subunits may be the basis of precipitability with McAb. The presence of the antigen in breast and gynaecological cancers correlated highly significantly with the oestrogen receptor expression. The antigen could thus be demonstrated in 10/12 oestrogen receptor positive but only 1/8 receptor negative breast carcinomas. The use of the McAb in immunohistochemistry facilitates the assessment of oestrogen receptor status of breast and gynaecological cancers, including the extent of heterogeneity within individual carcinomas.

**Characterisation of McAbs to pancreatic cancer cell structures**

R. Arndt, W.H. Schmiegel, H. Kalthoff, J. Gieseking, G. Skoppel, A. Ladak, V. Lampe & S. Ulrich

*Medical Clinic and Institute of Pathology, University of Hamburg, W Germany*

C540H5 (IgG2bk), C1N3 (IgG1K) and C1P83 (IgG1K) are McAbs generated by fusion of B-cells from Balb/c mice immunised with human pancreatic cancer cell lines (COLO 357, CAPAN 1). Antibody C540H5 was bound to pancreatic cancer cell lines (QGP-1, CAPAN-1, CAPAN-2, COLO 357, SW950), cholangio cancer cell line RPMI 7451 and bladder cancer cell line 647 V but only to 1/4 fibroblast cell lines in ELISA. Immunohistology revealed strong reactivity of C540H5 with 8/8 pancreatic tumours as well as with colon tumour, gall bladder tumour and breast cancer whereas there was a weaker reaction with 3/3 normal pancreas and normal liver tissue. Immunoprecipitation followed by SDS-PAGE and autoradiography showed that the antigen specifically bound by C540H5 is a protein with a mol. wt. of 125kD. C1N3 was specifically bound to 4/5 pancreatic cancer but not to various fibroblast and bladder cancer cell lines. Immunohistology revealed strong reactivity of C1N3 with 10/12 pancreatic tumours and no reaction with 3/3 normal pancreas tissues. C1P83 was specifically bound to pancreatic cancer cell line QGP-1 and cholangio cancer cell line RPMI 7451 and not to various fibroblast and bladder cancer cell lines. Immunohistology revealed strong reactivity of C1P83 with 6/6 pancreatic tumours but no reactivity with 3/3 normal pancreas tissues as well as with gall bladder, liver, colon, kidney and spleen.

**Clinical significance of *in vitro* maturation defect of monocytes in cancer patients**

Z. Rudolf, G. Sera & G. Krosl

*The Institute of Oncology, Ljubljana, Yugoslavia*

Defect of *in vitro* maturation of monocytes has been assayed in 70 cancer patients and in healthy donors. The mean value of mature cells as well as the mean value of maturation index (MI) was significantly lower in cancer patients when compared with control group ($P=0.001$). The mean value of MI was in 52 patients with malignant melanoma $8\pm6\%$, in patients with colorectal carcinoma $11\pm8\%$, while in healthy donors it was $44\pm14\%$. A correlation could be established between MI and the extent of disease. Patients in stage I displayed the mean value of 7.1%, in stage III (regional metastatic disease) the mean value was 5.2%, while in patients with disseminated disease (stage IV) the mean value was 3%. The maturation defect was associated with yet unknown serum factor, and the mean value of MI in autologous sera was decreased ($8\pm6\%$ *vs.* $6\pm5\%$). In a smaller group of patients the MI was being tested at different phases of the disease and the findings correlated with the response to treatment. A marked increase in MI values was noted in patients with complete response, while in patients with progressive disease the observed values of MI were markedly decreased.

**Criteria for the selection of McAbs suited for immunoscintigraphy**

K. Bosslet, G. Luben, H. Harthus, U. Rausch[1], J. Mollenhauer[1], H. Kern[1] & H.H. Sedlacek

*Research Laboratories of Behringwerke AG, and [1]Institute for Cell Biology, University of Marburg, FRG*

McAbs induced by immunisation with the PaTuII pancreatic adenocarcinoma cell lines were screened for specifiy using histology combined with the immunoperoxidase method on cryopreserved human pancreatic carcinoma, pancreatitis and normal pancreatic tissue. Five McAbs of the following specificity were evaluated for their capacity to localise in pancreatic carcinomas xenografted to nude mice.

The localisation indices of the $^{131}$I-labelled McAbs were determined according to *Br. J. Cancer*, (1981), **44**, 91. Despite the fact that the antigens recognised by the different McAbs are expressed at similar quantities ($2\text{–}5\times10^5$ antigen molecules/cell) on the plasma membrane of *in vitro* cultured pancreatic carcinoma cell lines, the localisation indices varied from 1 (for 227/19, no localisation) up to 7 (250/183). In this respect, the affinity of the McAbs and antigen shedding are important contributory factors.

| *McAb* | *Pancreatic carcinoma* | *Normal pancreatic tissue* | *Reactivity with nude mouse tissue* | *MW of antigens detected* |
|---|---|---|---|---|
| 227/18 | ++ | ++ | — | 130 kD |
| 227/19 | ++ | +macrophages | — | 43 kD |
| 406/14 | ++ | (+)/+ducts | — | 200 kD |
| 431/13 | ++ | — | — | 180 kD |
| 250/183 | ++ | — | — | 180 kD |

**Radiolabelled McAbs for tumour immunodetection: Comparison between two indium chelating agents**

J.Y. Douillard, J.C. Saccavini, J.F. Chatal & B.P. Le Mevel

*INSERM U 211, UER Medecine, Nantes, France*

Indium-labelled McAbs with tumour specificity have already been utilised to localise tumours by immunoscanning. Non-specific accumulation in the liver was consistently noticed and considered as a major problem for liver metastasis detection.

In order to reduce the non-specific binding of In-111 McAb to normal tissues, two chelating agents, DTPA or TTHA, were comparatively used to bind In-111 to gastrointestinal tumour-specific McAb. Biodistribution was analysed by sacrificing nude mice bearing human colon carcinoma at different time points after injection of In-111-labelled specific McAb. Results obtained at all the times of the experiments (1, 5 and 7 days) show quite similar antibody distribution independently of the chelating agent used for In-111 labelling. Non-specific accumulation occurred mainly in liver, spleen, kidney, skin and bone, with a slight advantage to DTPA over TTHA resulting in higher tumour/normal tissue ratios. Tumour/liver, T/spleen, T/kidney, T/skin and T/bone ratios were below 1, reflecting the non-specific binding of In-111 McAb to normal tissues, for both DTPA and TTHA In-111-labelled antibodies. Non-specific binding of In-111-labelled McAb to normal tissues does not favour the use of In-111 as a radionuclide for tumour detection with radiolabelled McAb in cancer patients.

**Improved tumour localisation using subtraction of indium-labelled specific and gallium-labelled non-specific antibody**

H. Haisma, W. Goedemans & J. Hilkens

*Antoni van Leeuwenhoekhuis, Plesmanlaan 121, Amsterdam, The Netherlands*

A murine McAb, reactive with human mammary tumours, was radiolabelled with In-111 and injected into nude mice bearing human tumour xenografts, together with Ga-67 radiolabelled non-specific Ig. Tumours could be visualised clearly with the In-labelled specific antibody, but an improved localisation was obtained when the image was subtracted with the image of the Ga-labelled non-specific antibody. Tumour to tissue contrast was improved from 2.9 to 7.6 after subtraction.

In-111 radiolabelled antibodies showed higher tumour to tissue ratios than I-123 radiolabelled antibodies. It was shown that two biochemically related isotopes, In-111 and Ga-67, could be imaged at the same time using a gamma camera in dual isotope mode. Overlap of each isotope in the other isotope channel was $<20\%$. Indium and gallium-labelled antibodies could be injected simultaneously and images could be produced up to 5 days after injection.

This technique offers advantages over previously used subtraction methods which use Tc-99m albumin and pertechnetate for subtraction of blood pool activity of the radiolabelled antibody. The antibody used for subtraction is injected simultaneously with the specific antibody and does not have to be injected before each scan. Furthermore, the use of a second antibody seems more appropriate, as the distribution of this antibody in non-malignant tissue is identical to the distribution of the specific antibody.

### WAYS TO IMPROVE LOCALISATION
(Chairman J.P. Lavender)

**Radioimaging of melanoma lesions with McAbs to a human high molecular weight melanoma associated antigen**

S. Ferrone, P. Giacomini, L. Callegaro, U. Rosa & G. Buraggi

*Department of Microbiology and Immunology, New York Medical College, Valhalla, NY, USA*

The high mol. wt. melanoma associated antigen (HMW-MAA) has the following properties: (i) it is expressed by at least 90% of melanoma lesions, is not detectable in normal tissues except for hair bulbs and is found in minute amounts in serum; (ii) it has a density of $\sim 2 \times 10^6$ antigenic sites/cell on the melanoma cell line Colo 38; (iii) it is not susceptible to antibody mediated modulation, even if the incubation is made in the presence of antimouse Ig xenoantibodies; (iv) anti HMW-MAA McAbs 149.63, 225.28S and 763.74T have high affinity since their association binding constants were found to be $1.01 \times 10^8$ moles $\times l^{-1}$, $0.98 \times 10^8$ moles $\times l^{-1}$ and $1.26 \times 10^8$ moles $\times l^{-1}$, respectively. Injection of $^{131}$I-McAb 225.28S into patients with melanoma could visualise malignant lesions within 60 min, but was also associated with accumulation of radioactivity in liver, spleen and bone marrow. This background is likely to be caused by the uptake of the antibody (complexed with the circulating HMW-MAA?) by Fc receptor bearing cells, since it was markedly reduced when $Fab_2$ fragments were injected into patients. $Fab_2$ fragments of the McAb 225.28S labelled with $^{125}$I, $^{131}$I, $^{111}$In and $^{99m}$Tc were effective in visualising malignant lesions in 11 of the 18 patients investigated. Lesions with a diameter of at least 2.5 cm could be detected. These results suggest that the HMW-MAA is a useful marker for radioimaging, provided that $Fab_2$ fragments are utilised.

### The use of second antibody to improve selective localisation of tumours: Animal model studies

K.A. Chester, R.H.J. Begent, B. Pedley, P.A. Keep, J.A. Boden, F. Searle & K.D. Bagshawe

*Department of Medical Oncology, Charing Cross Hospital, London, W6 8RF, UK*

The potential of tumour directed (antitumour) antibodies for localisation and therapy of cancer is limited by the relatively large proportion of administered antibody which remains in the normal tissue. Second antibody, directed against the antitumour antibody, may be used as a tool to facilitate clearance of non-tumour associated antitumour antibody.

The effect of second antibody on tumour and normal tissue localisation of $^{125}$I-labelled goat antibody directed against carcinoembryonic antigen (antiCEA) was investigated in nude mice bearing human colorectal carcinoma xenografts. Second antibody clearance of $^{125}$I-labelled mouse monoclonal antibody directed against human chorionic gonadotrophin was studied in rabbits.

Results showed that second antibody, administered 6 h after antiCEA at 10 times the dose, was able to cause a 10-fold reduction in circulating antiCEA and an 8-fold increase in tumour: blood ratios in mice. In rabbits a 40-fold reduction of control values for circulating monoclonal antibody was achieved and there were no clinical or histological indications of immune complex-mediated illness.

These results indicate that second antibody could be used to improve tumour discrimination for location and therapy of cancer.

### The use of second antibody to improve selective localisation of tumours: Clinical studies

R.H.J. Begent, A.J. Green, K.A. Chester, P.A. Keep, F. Searle & K.D. Bagshawe

*Department of Medical Oncology, Charing Cross Hospital, London, W6 8RF, UK.*

Localisation of cancer by external scintigraphy after i.v. injection of radiolabelled anti-tumour (first) antibody is hampered by the high proportion of antibody which persists in normal tissues. We have previously shown in animals that non-tumour-bound first antibody could be cleared from normal tissues by second antibody directed against the anti-tumour antibody. The second antibody could either be free or entrapped in liposomes. Eight patients have received liposome entrapped second antibody (LESA): in 5, goat antiCEA was the first antibody, and in 3, mouse monoclonal antibody (17-1A) was used. Second antibodies were horse and rabbit respectively. Good clearance of first antibody was seen in all those with adequate doses of LESA. Tumour images were enhanced in this way. Fourteen patients have received free second antibody (rabbit anti-mouse). Clearance of first antibody was achieved for 5 patients receiving 17-1A antibody and 5 with mouse monoclonal antibody to HCG. Tumour images were thus enhanced. Two McAbs (antiCEA and antiAFP) failed to clear with free second antibody. When favourable combinations of first and second antibody are used either LESA or free second antibody can enhance tumour imaging and may have application in improving the therapeutic ratio of antibody directed therapy of cancer.

### Experience with $^{123}$I-labelled McAbs

K.E. Britton & M. Granowska

*Department of Nuclear Medicine, St Bartholomew's Hospital, London, EC1, UK*

Tumour associated McAbs, may be labelled with $^{123}$I using the mild iodogen technique which by direct immunoassay causes no reduction in immunoreactivity. Since the majority of uptake of labelled McAbs *in vivo* is completed by 24 h, the use of short-lived $^{123}$I (13.2 h half life, $\gamma$ energy 0.159 MeV, no beta radiation) is feasible. The maximum uptake of the injected McAb is of the order of 7%, usually 1–2%, thus initially the blood and tissue background is high. Two approaches to imaging may be compared: early imaging with $^{123}$I for high count rate and good statistical data, coupled with a technique for background correction by subtracting an early blood pool image from the later blood pool and uptake images using a repositioning protocol for the patients and their computer images together with a temporal change detection algorithm; or late imaging with a long-lived radionuclide such as $^{131}$I with improved target to noise ratios but at the expense of poor sensitivity and poor statistics. Over 10 times the count rate is obtained using $^{123}$I over $^{131}$I for the same administered dose and for a lesser radiation dose. In one child with neuroblastoma imaged with $^{131}$I UJ13A, the tumour was evident only after 5 days, whereas in the same child imaged with $^{123}$I UJ13A, the tumour was well visualised at 24 h even without computer analysis. $^{123}$I labelled HMFG2 McAb has been used successfully to image known ovarian cancer and its spread, with a 90% correlation with the surgical findings in a prospective study. McAbs labelled with $^{123}$I are excellent for the early imaging of the distribution and response to chemotherapy of malignant disease, for counts count in nuclear medicine.

### 111-Indium-labelled antibodies for tumour detection

D.S. Fairweather, A.R. Bradwell, N. Baggett, S. Chandler[1] & P.W. Dykes

*Departments of Immunology and Chemistry, University of Birmingham, and [1]Department of Nuclear Medicine, Queen Elizabeth Hospital, Birmingham, UK*

Tumour detection using radiolabelled antibodies has been shown to be a sensitive and specific technique. $^{131}$Iodine is frequently used as the radiolabel but produces high energy gamma and beta emissions which are disadvantageous. $^{111}$Indium, by contrast, has a much more suitable gamma ray and is known to be a stable cell label. We have labelled antibodies to carcinoembryonic antigen (CEA) with $^{111}$In and used them for tumour detection in comparison with $^{131}$Iodine labelled antiCEA, (131-I-aCEA). DTPA was covalently coupled to sheep antiCEA using a mixed anhydride. 74 MBq of $^{111}$In chloride was neutralised with 0.3 M acetate (pH 6.5), mixed with 0.8 mg of modified antibody, and left at 4°C overnight. Unbound $^{111}$In was removed by gel-filtration. Labelling efficiency was 50–75% giving a specific activity of up to 74 MBq/mg protein. Eleven patients with CEA producing tumours were studied; 5 had repeat scans using the same dose of 131-I-aCEA. $^{131}$I did not accumulate in any organ and was rapidly excreted in the urine. With Indium, 20% of the injected dose accumulated in the liver, 10% was excreted in the first 24 h and 3%/day thereafter. Whole body absorbed radiation doses were similar for both isotopes but count rates with $^{111}$In were 3-fold higher. The 11 patients had 31 potential tumour areas. Twenty-eight were positive by the $^{111}$In scans and 25 positive by a combination of conventional clinical tests. The 5 patients scanned with both isotopes had 15 potential tumour areas. 13 were detected with $^{111}$In and 8 with $^{131}$I. The $^{111}$In scans also achieved higher statistical significance. Despite similar dosimetry, $^{111}$In is clearly a superior isotope to $^{131}$I for scanning. This is due to higher count rates and prolonged tumour residence of Indium giving improved picture contrast.

### Improved radiolocalisation using other than intravenous routes of antibody administration

A.A. Epenetos

*On behalf of Hammersmith Oncology Group, Royal Postgraduate Medical School, Hammersmith Hospital, London, and the Imperial Cancer Research Fund, London, UK*

Using a paired antibody technique (including specific and non-specific radio-iodinated antibodies) we showed that although good tumour to non-tumour (T:NT) ratios could be obtained in patients with various epithelial neoplasms, the absolute amount of specific radiolabelled antibody targeted to tumours was small ($\sim$0.01% of injected amount $g^{-1}$ tumour 1 day post-administration).

Thus, we investigated other routes of antibody administration. We found improved T:NT and significantly higher absolute antibody uptake when radiolabelled antibodies were given i.p. to patients with ovarian cancer, intralymphatically to patients with lymph node metastases secondary to cervical cancer, intrapleurally to patients with intractable pleural effusion, intrapericardially to patients with pericardial effusion and impending cardiac tamponade, and as an intra-arterial infusion in patients with gliomas.

These routes of antibody administration offer the advantages of diminished catabolism and dehalogenation of radio-iodinated antibodies and high antibody concentration for longer periods of time in the vicinity of tumours. In this way, radiolabelled antibodies can be used successfully for the diagnosis and possible therapy of some forms of malignant disease.

### OTHER POTENTIAL DIAGNOSTIC USES OF ANTIBODIES

(Chairman A.J. Munro)

### Gadolinium-DTPA as a contrast agent in magnetic resonance imaging

D.H. Carr[1], J. Brown[2], G.M. Bydder[1], I.R. Young[3] & R.E. Steiner[1]

[1]*Department of Diagnostic Radiology and* [2]*Department of Clinical Pharmacology, Royal Postgraduate Medical School, Hammersmith Hospital, London, and* [3]*Picker International, Wembley, Middlesex, UK*

Forty-six patients were examined on a 0.15 Tesla superconducting imaging system before and after intravenous administration of Gd-DTPA (Schering) in a dose of 0.1 mmol kg$^{-1}$. The 46 patients included 20 with malignant cerebral tumours, 5 with non-malignant disease of the brain, 12 with tumours of the liver (benign and malignant), 5 with renal tumours, as well as patients with tumours in the bladder, pancreas, femur and mediastinum. All 20 patients with cerebral tumours showed enhancement. In 14/20 cases, contrast enhancement allowed the differentiation between tumoral and peritumoral oedema to be made. Enhancement was noted in all the other patients with the effects being most marked on inversion recovery scans. No significant abnormality was noted in any haematological or biochemical parameter after Gd-DPTA administration. Intravenous Gadolinium-DPTA promises to be a useful adjunct in magnetic resonance imaging.

### The measurement of regional tissue function using positron emission tomography

R.P. Beaney, D.J. Brooks, K.E. Halnan & T. Jones

*MRC Cyclotron Unit, Hammersmith Hospital, London, W12 0HS, UK*

With the advent of positron emission tomography it has become possible to measure the regional tissue concentration of a positron emitting radionuclide in absolute units (Bq ml$^{-1}$). Using the sequentially inhaled tracers $C^{15}O_2$ and $^{11}CO$ it is possible to measure regional blood flow and blood volume in man non-invasively. The uptake of any blood-borne tracer is governed not only by regional tissue perfusion (supply), but also by the regional extraction by the tissue. Consequently by measuring both the regional tissue uptake of a tracer and regional blood flow one can derive the regional extraction of the tracer by means of positron emission tomography. It is relatively easy to correct for the blood pool by using the regional blood volume data obtained after the inhalation of $^{11}CO$.

To date most tumour PET studies have dealt with the tissue extraction of molecular $^{15}O_2$, the glucose analogue $^{18}$F-2-deoxyglucose and the cation 82-Rubidium. The same technique, however, has been used to measure the extraction of $^{11}$C-Albumin and $^{11}$C-labelled BCNU in brain tumours and can easily be extended to measure the tissue uptake of *suitably labelled* McAbs.

### Anti-CEA gamma camera scintigraphy with different antibodies and radionuclides

P.O. Schnell, S. Hammarström, S. von Krusenstierna, S.A. Larsson, G. Lundell & B. Wahren

*Karolinska Hospital, Stockholm, Sweden*

Our earlier studies with goat antibodies labelled with $^{131}$I or $^{123}$I resulted in detection of 50–60% of known gastrointestinal tumours.

In some of the examinations we have used SPECT with a rotating gamma camera in combination with conventional gamma camera scintigraphy.

In an attempt to improve the results we have used, in the last year, monkey anti-CEA and monoclonal mouse anti-CEA (McAb 38S1). The results are seen in the following table:

| | *No. of tumours* | *% detected* |
|---|---|---|
| Monkey (macaca irus) anti-CEA-$^{131}$I | 30 | 80 |
| Mouse McAb 38S1-$^{131}$I | 30 | 73 |

The change of antibodies has improved the detectability of human CEA-producing tumours in our studies to 70–80%. A new method developed by Dr E. Sundrehagen, Radiumhospitalet, Oslo, Norway, to label IgG with $^{99}$Tc$^{m}$ has been tested *in vitro*. Preliminary results have given a labelling yield of ~85%.

### Immunoscintigraphy with $^{99m}Tc$ labelled $F(ab')_2$ fragments of a melanoma associated McAb

G. Paganelli & P. Riva

*Department of Nuclear Medicine, Ospedale Generale Provinciale "M. Bufalini", Cesena, Italy*

Twelve patients with stage III or IV malignant melanoma were studied with $^{99m}Tc$ labelled $F(ab')_2$ fragments of a melanoma associated McAb (Tecnemab-1, Sorin-Biomedica, Italy). The amount of administered antibody ranged from 100 to 200 μg of protein labelled with 8 mCi (296 MBq) $^{99m}Tc$. No adverse reactions were observed.

Conventional clinical investigation revealed 68 metastatic sites. Of these, 49 (72%) were detected by immunoscintigraphy. Immunoscintigraphy also showed 31 further positive scans, 11 of which were confirmed by further investigations. Patients were scanned at 4, 8 and 24 h after i.v. injection, the best images being obtained at 8 and 24 h.

This work is part of a multicentre clinical trial "Immunoscintigraphy of malignant melanoma" conducted by the National Research Council, Special Project on Biomedical and Clinical Engineering.

### Clinical use of radiolabelled tumour-associated antibodies: State of the art and future prospects

N. Pateisky, K. Philipp & J. Burchell

*Department of Obstetrics and Gynaecology, University of Vienna, and ICRF, London, UK*

The aim of this study was to examine the clinical value of radioimmuno-localisation in patients with carcinoma of the ovary, cervix uteri and breast using the tumour-associated McAb HMFG2.

Fifteen patients were studied and the results from antibody scans were compared to surgical findings. $^{123}I$ labelled antibody was given to patients either i.v. or s.c. Both routes of administration were found to be of clinical value. The i.v. route was useful in establishing the diagnosis and in the follow-up of patients, whilst the s.c. route was useful in the pre-operative assessment of lymph node status. The number of patients studied so far is small but the accuracy of this test appears to be high.

In 1969 Gitsch and co-workers introduced intra-operative lymphoscintigraphy in an attempt to improve the accuracy of lymph node dissection in patients with cervical cancer. It is possible that the accuracy of lymphoscintigraphy could be improved by using radiolabelled McAbs such as HMFG2.

### THERAPEUTIC APPLICATIONS OF ANTIBODIES *IN VITRO*

(Chairman D.A.G. Galton)

### Antibody-ricin conjugates: A method of conjugation that blocks the galactose recognition site on the B-chain

B. Foxwell, P. Thorpe & W. Ross[1]

*Imperial Cancer Research Fund, Lincoln's Inn Fields, London, WC2A 3PX, and [1]Institute of Cancer Research, Fulham Road, London, SW3, UK*

Ricin, the toxic lectin from castor bean, is a glycoprotein which consists of two polypeptide chains, A and B, linked by a disulphide bond. Ricin exerts its toxic action by binding to cell surface oligosaccharide structures via a galactose recognition site on the B-chain. This is followed by the internalization of at least the A-chain which subsequently catalytically inactivates 60S ribosomal subunits.

Recently, attempts have been made to target ricin to malignant cells by covalently linking it to McAbs directed against neoplastic tissues. Two approaches of antibody-ricin conjugate construction have so far been used. One method is to link the isolated A-chain by a disulphide bond to the antibody. Some conjugates of this type have proved capable of highly specific toxicity to cells bearing the appropriate antigen. Unfortunately many of these A-chain conjugates are poor cytotoxins probably because, lacking the B-chain, they are unable to penetrate cells as efficiently as native ricin.

A second approach is to link the intact toxin to the antibody; a method has been developed that produces a blockade of the galactose binding site on the B-chain and so prevents non-specific toxicity. Such conjugates have been found in tissue culture test systems to have a $10^4$-fold greater toxicity towards cells bearing the target antigen than to those without it. The conjugates are also highly effective at killing cells in murine tumour models. They were able to kill 99.99% of i.p. administered tumour cells when injected 24 h later i.p. I.v. injection reduced effectiveness by 10-fold.

### Use of McAbs for removal of unwanted cells from harvested bone marrow (BM)

J.M. Goldman

*MRC Leukaemia Unit, Royal Postgraduate Medical School, London, UK*

Immunological techniques involving the use of McAbs can be used *in vitro* either to remove neoplastic cells from harvested BM before autografting or to deplete allogeneic BM of T-lymphocytes in the hope of suppressing or preventing graft-versus-host disease (GVHD). One may rely either on the opsonizing capacity of a non-complement-fixing IgG McAb or on the direct complement-dependent cytotoxicity *in vitro* of an IgM McAb.

*Autografting after purging marrow*: The use of McAbs to purge BM in acute leukaemia depends on the assumption, which may not be valid, that normal and leukaemic stem cells are antigenically distinguishable. The J5 McAb (directed against common-ALL antigen) has been used to treat autologous BM before autografting for patients with ALL in second remission. The development of myeloid McAb should permit this technique to be applied in AML.

*T-cell depletion before allogeneic BM transplantation*: The Royal Free Hospital team has recently reported the use of two anti-T cell McAbs in combination followed by treatment with rabbit complement to remove T-lymphocytes from donor BM. This technique appears to reduce very substantially the incidence and severity of GVHD. At the Hammersmith we have used a human complement-fixing IgM McAb designated Campath-1 to treat BM *in vitro*. The speed of engraftment of BM from HLA-identical sibling donors resembles that of untreated BM and the severity of GVHD seems to be reduced; engraftment of mis-matched BM cells may however be compromised by such T-cell depletion.

### Ways to improve the *in vitro* cytotoxicity of immunotoxins

A. Frankel, D. Ring, L. Greenfield & M. Bjorn

*Cetus Corporation, Emeryville, California, USA*

Seventy McAbs to human breast cancer-associated antigens were linked by SPDP to ricin toxin A chain and tested on three breast cancer cell lines and one normal fibroblast cell line for their ability to inhibit protein synthesis. Factors that predicted *in vitro* potency were IgG class, cell surface binding, high affinity and high antigen copy number per cell.

Several of the McAbs were linked by 2-iminothiolane or MBS to ricin A chain. Only disulfide linkages to ricin A chain yielded active immunotoxins.

Several of the McAbs were linked by SPDP or MBS to diphtheria A chain, CRM45, MSP (Greenfield, L., 1983, *Proc. Natl Assoc. Sci.*, **80**, 5683) and MSP-SA (spacer arm) and tested for cytotoxicity. Only the genetically engineered MSP-SA containing the diphtheria hydrophobic region and a spacer arm was active *in vitro*. Another toxin moiety, PAPII, was conjugated via SPDP or 2-iminothiolane to McAbs and yielded conjugates less active than ricin A chain.

### T-cell depletion of human bone marrow using T101 A-chain immunotoxin

G. Laurent, P. Poncelet, A. Fauser[1], J.M. Derocq & F.K. Jansen

*Centre de Recherche Clin-Midy-SANOFI, Montpellier, France, and [1]Medizinische Universitat Klinik, Freiburg, FRG*

Acute graft versus host disease (GVHD) has, for years, been the major cause of morbidity during the initial one to two months following allogeneic bone marrow transplantation (BMT). Many experiments in animals, as well as a number of human trials, have shown that it could be greatly reduced if mature T lymphocytes were removed from the donor marrow. In an attempt to abrogate acute GVHD, anti-T cell immunotoxins (IT) offer an efficient option. An anti-human T-cell A-chain-IT was prepared by coupling ricin A-chain with the McAb T101, and evaluated for both efficacy and tolerance.

Under defined conditions, T101-A-IT allowed: (1) a reduction of the PHA, con A and mixed lymphocyte culture to levels below background; (2) a cyto-reduction of more than 2 logs of the mature T lymphocytes evaluated in a clonogenic assay.

With regard to tolerance, no toxicity of stem cells could be found using CFU-GM, BFU-E and CFU-GEMM assays up to the concentration of $10^{-8}$ M. These results show that the use of T101-A-IT is a simple, reliable, highly efficient and safe approach to the *in vitro* treatment of bone marrow in allogeneic transplantation.

### Activity and specificity of abrin and ricin immunotoxins

Ø. Fodstad, A. Godal & A. Pihl

*Norsk Hydro's Institute for Cancer Research and the Norwegian Cancer Society, Oslo, Norway*

Conjugates of abrin and ricin with antibodies directed against human melanoma-associated antigens (250 K and p 210) were prepared. The conjugates were tested against a panel of melanoma cell lines with different degrees of expression of the antigens.

The abrin conjugates exhibited greater toxicity and specificity than the corresponding ricin conjugates. The toxicity, as measured by the inhibition of cellular protein synthesis, was related to both the degree of expression of the appropriate antigen and to the different sensitivities of the cells to the unconjugated toxin. Addition of free antibody strongly reduced the toxicity of the conjugates to antigen positive cells, but had no effect on non-melanoma control cells.

### THERAPEUTIC APPLICATIONS OF ANTIBODIES *IN VIVO*

(Chairman A.A. Epenetos)

### The cellular expression of a heavy chain linked idiotypic determinant recognised by a monoclonal anti-idiotypic antibody in a lymphoma patient and his relatives

J.A. Habeshaw, L.C. Walker & S Dhut

*ICRF Department of Medical Oncology, St Bartholomew's Hospital, London, UK*

McAbs reactive with idiotypic determinants of a paraprotein JS IgG1$\lambda$ were characterised by their reactivity with Fab, and isolated H chain and L chain determinants. Three McAbs were reactive with a conformational determinant requiring both heavy and light chain association, 6 identified heavy chain determinants, and 7 reacted with $\lambda$ chain. A McAb 2H3.D8 reactive with heavy chain idiotype was used to detect expression of this idiotype by tumour cells from the patient J.S., and by B lymphocytes in the blood of his mother, his two brothers, and his daughter. The idiotype detected by 2H3.D8 was expressed on 0.15% of maternal B cells and was found in lower or similar frequency in normal controls. In the patient 72% of circulating B cells expressed this determinant. In both brothers, and in the daughter, 2H3.D8 idiotype was expressed on 10% ($\pm$2%) of circulating B cells. Similar values for expression of this idiotype were obtained from studies of the serum Ig in the patient's family. These results suggest the occurrence of heavy chain linked 2H3.D8 idiotype is a genetically determined dominant characteristic inherited through the patient's father. Cross reactivity of McAb 2H3.D8 was not observed in panel testing of over 70 other paraproteins.

### Radiolabelled antibodies: Dosimetry studies

M.J. Myers & G.R. Hooker

*Department of Medical Physics, Hammersmith Hospital, London, UK*

I.v. injection of radiolabelled antibodies has been shown, by post-operative sampling, to yield too low an absolute uptake of activity by the tumour for therapeutic applications. Therapy has been directed instead to the treatment of tumours of peritoneal, pleural and pericardiac cavities where the radiolabelled antibody can be administered into an enclosed space and tumour uptake relative to normal or whole body uptake improved to practical levels. In order to calculate the radiation dose received by the tumour and by normal tissues it is necessary to know the activity and its residence time in a particular volume of tissue. The therapeutic study is therefore divided into two phases. The first employs a relatively small amount of activity (usually 1 mCi of $^{131}$I) attached to the antibody. Sequential quantitative whole body imaging using a scintillation camera/computer system gives the time course and distribution of the administered activity. Other imaging modalities such as X-ray CT and ultrasound aid the estimation of the volume of distribution in the tumour (this is the least accurately measured parameter and may lead to gross errors in the final dosimetric calculations). Based on these findings and on the dose estimated to be received by normal organs and the whole body a therapeutic dose (of the order of 20–50 mCi) can be administered as phase two of the study in the same way as in phase one. The localisation and time course of the therapeutic activity can be monitored only approximately to confirm the previous estimated radiation dose. The results of six studies using $^{131}$I labelled HMFG1 and HMFG2 tumour-associated antibodies are outlined and the method of dose calculation detailed.

### McAb targeting anti-cancer drugs

R.W. Baldwin

*Cancer Research Campaign Laboratories, University of Nottingham, UK*

Tumour localizing McAbs have considerable potential for targeting chemotherapeutic agents since they provide a means for the selective delivery of drugs to a local tumour, or more particularly, to metastases. There are several inter-related steps in designing drug-antibody conjugates for therapy. This includes selection of McAbs which localize in tumours ideally with little or no uptake into normal tissues. Also for effective delivery of drugs linked to antibody, conjugates should uniformly penetrate regions of the tumour which are contributing to its progressive growth. The amount of antibody which can be deposited in a tumour must also be sufficient to provide targeting of appropriate levels of drug.

There are several factors to be taken into account in designing drug-antibody conjugates depending upon the final stage involved in drug transfer to tumour cell. One approach is to covalently link antibody to drug so that after antibody binding to the tumour cell it will internalize as an intact conjugate so allowing the drug moiety to exert its cytotoxic effect. Antibodies may also be used to target drugs to tumour so that after localization the drug moiety may be released and function as free drug.

These concepts and development will be illustrated by considering the tumour localizing properties of McAb 791T/36. The construction and evaluation of 791T/36 antibody conjugate will be considered with respect to chemotherapeutic agents including methotrexate, daunomycin, vindesine and interferon as an immunomodulating agent.

### Boron-10-labelled antibodies for neutron capture therapy

R.G. Fairchild, J.J. Elmore, D.C. Borg, P. Micca, D. Gabel & B.H. Laster

*Medical Department, Brookhaven National Laboratory, Upton, New York, USA*

If they were truly tumour specific, McAbs would provide unique access to various tumours. In fact, therapeutic application of radiolabelled antibodies delivers significant doses to normal cell pools which compete for the McAbs. This problem can be circumvented by targeting stable (non-toxic) isotopes to tumour *via* McAbs, which can subsequently be activated by external radiation beams. Competing normal cell pools can then be excluded from the treatment volume. Such a technique is available via the $^{10}B(n,\alpha)^{7}Li$ reaction. Calculations show that therapeutic amounts of $^{10}B$ can be delivered assuming $10^3$ $^{10}B$ atoms per McAb, and $10^6$ antigen sites per cell ($\sim 15\,\mu g$ $^{10}B$ per g tumour). Direct conjugation of such amounts of B to McAbs is difficult. We are using dextrans as "bridges" to enhance the carrying capacity of McAbs. Experiments to date indicate that ~1,000 boron atoms can be attached to McAbs via dextran bridges while still retaining biological activity. Three to five dextrans (40k dalton) have been successfully attached to anti-IgM antibodies. These are subsequently reacted with amine derivatives of decachlorocarboranes, so that an average of ~20 cages are bound per dextran. These conjugates have been demonstrated to be biologically active *vs* IgM, with affinity chromatography (sepharose-bound IgM). Preliminary studies with the U698 cell line (which has IgM on the cell membrane) have shown that following a 30 min incubation period with the boronated McAbs described above, significant binding to the U698 cells was achieved. Direct boron assay indciated $\sim 5\,\mu g$ boron $g^{-1}$ cells. Collaborative studies are underway with Dr Soldano Ferrone, to prepare similar McAbs directed to human melanoma.

Research supported by NIH Grant No. CA32920 and the U.S.D.O.E.

### McAbs of therapeutic potential which interact with host effector systems

M. Clark, S. Cobbold, G. Hale & H. Waldmann

*Department of Pathology, University of Cambridge, UK*

Rat McAbs made against various subpopulations of mouse and human haemopoietic cells have been tested for their ability to eliminate these cells both *in vitro* and *in vivo* using host effector systems. Many rat IgM and IgG antibodies against human cells are effective at promoting human complement mediated lysis and an example of such an antibody CAMPATH1 (Hale *et al.*, 1983, *Blood*, **62,** 873), with specificity for human lymphocytes is presently being tested clinically for its ability to prevent GvHD in bone marrow transplantation. Complement mediated lysis with IgG antibodies of less potency can be improved by preparing the monovalent antibody (Cobbold & Waldmann, 1984, *Nature*, in press).

In tests of antibody dependent cell-mediated cytotoxicity using human lymphocytes as effectors no killing was observed with 18 IgM, 23 IgG2a or 4 IgG2c antibodies but all 21 IgG2b antibodies tested gave killing even at high dilution (Hale *et al.*, 1984, *Biochem. Soc. Trans.*, in press).

Various isotypes of rat antibodies against mouse lymphocyte subpopulations were tested for their ability to suppress *in vivo* immune responses and graft rejection. Only antibodies of the IgG2b isotype were found to work (Cobbold *et al.*, 1983, *Mol. Biol. Med.*, **1,** 285). It seems clear from these results that antibody isotype as well as specificity will determine the therapeutic potential of McAbs.

**Therapeutic use of monoclonal anti-idiotype antibodies against B-cell lymphoma**

E.M. Rankin, A. Hekman, R. Somers & W.W. ten Bokkel Huinink

*The Netherlands Cancer Institute, Plesmanlaan 121, Amsterdam, The Netherlands*

Two monoclonal anti-idiotype antibodies have been used for the treatment of advanced centrocytic lymphoma, antibody T2 for patient Top, and antibody K1 for patient Kos. Both antibodies were of the IgG2a subclass, were cytotoxic with rabbit but not with human complement, and did not modulate the antigen *in vitro*. Patient Top had $10 \times 10^9$ malignant lymphocytes in the blood, and a negligible amount of free idiotype. A variety of different schedules of administration, daily dosage doubling, hourly dosage doubling and continuous low-dose infusion were tried, all resulted in a temporary fall in the level of lymphocytes. At the end of the continuous infusions, free antibody was detectable in the serum, and cells in blood, bone marrow, lymph node and ascites were coated with antibody. $^{111}$Indium oxine labelled lymphocytes were cleared from the circulation which was rapidly populated with unlabelled cells. The S-phase cells in the blood and $^3$H-thymidine uptake did not alter during treatment. Lytic cells, a sign of necrosis, increased in the lymph nodes from 0 to 25%. Monocyte activity improved during treatment.

Patient Kos had large amounts of free idiotype in the circulation. This was removed by antibody K1, and coating of cells with K1 in blood, lymph node and ascites was demonstrated, excess K1 was detectable in the serum at $20\,\mu g\,ml^{-1}$.

Both patients have had a minimal response to treatment. 3.8 g T2, and 5.9 g K1 were given without any toxicity. No modulation of the antigen, and no antibodies to the mouse Ig were seen.

**Treatment of chronic lymphatic leukaemia with monoclonal anti-idiotypic antibodies**

P.J.A. Capel, F.W.M.B. Preijers & W. Allebes

*Department of Nephrology, Radboud Hospital, Nijmegen, The Netherlands*

A McAb of the IgG1 subclass reactive with the idiotype of the leukaemic B-cells of a BCLL patient was raised. This antibody did not show any cross reactivity with other cells or tissues, and in total 40 grams of anti-idiotypic antibody was purified from ascites. Because human Fc receptors are polymorphic (only 70% of normal individuals can react with murine IgG1), the patient's Fc receptors were typed and showed to be reactive with murine IgGl. The patient had a high level of free circulating idiotype ($150\,\mu g\,ml^{-1}$) and upon *in vivo* treatment the free idiotype was cleared without any signs of serum sickness or other side effects. After the removal of free idiotype a strong tumour reduction was observed. The swollen lymph nodes decreased to a normal size and the spleen, with a weight of ~4 kg, reduced 20% in size. *In vivo* a moderate modulation of the antigen was present but the idiotype was re-expressed within 16 h. During repeating treatments the *in vivo* modulation became more pronounced and the therapy lost its effectiveness. Due to antigen modulation immunotoxins might be effective in such case. Therefore ricin A chain was conjugated to the anti-idiotype and tested *in vitro* for specific cell killing. The specific toxicity of the immunotoxins was too low to consider any therapeutical use. This low specific toxicity was most likely due to a low idiotype expression of the B-CLL cells.

We may conclude that murine IgG1 anti-idiotypic antibodies are effective *in vivo* without any side effects even in the presence of high levels of free idiotype, but due to antigen modulation this type of therapy was only partially effective.

**Cell-mediated lysis of glioma cells directed by McAbs**

Th. Bilzer, D. Stavrou[1] & W. Mellert[1]

*Lehrstuhl für Allg. Pathol. und Neuropathol., & [1]Abt. Funktionelle Pathol., Institut für Tierpathologie, University of Munich, W Germany*

A McAb (14AC1) has been used for directing T cell-mediated antibody-dependent cytotoxicity (ADCC) against the syngeneic glioma cell line 79FR-G-41, derived from a N-methyl-N-nitrosourea induced rat astrocytoma. Hybridomas were established by fusion of the non-producing X63-AG8.653 mouse myeloma line and spleen cells from BALB/c mice immunized with 79FR-G-41 cells (Stavrou *et al.* (1983) *Eur. J. Cancer Clin. Oncol.*, **19**, 1439). Products of the 14AC1 clone show specific binding avidity and are characterized as belonging to the IgG2 isotype.

T cells have been enriched from thymus or peripheral blood of normal syngeneic Fischer rats (F344). Effector cells were non-adherent, non-phagocytic and surface-Ig negative. For ADCC glioma cells as well as syngeneic brain cells were triggered with serum-free hybridoma supernatants. In comparison with natural T cell-mediated cytotoxicity effector cells revealed a 50% increase of reactivity against antibody-coated glioma cells, whereas no cytotoxicity could be induced by coating the brain cells.

### A model for cell killing using radiolabelled antibodies

A.T.M. Vaughan, A.R. Bradwell, J. Taylor & P.W. Dykes

*Department of Immunology, University of Birmingham, UK*

A nine parameter model for the killing of tumour cells using radiolabelled antibodies has been developed. Included are physical variables such as isotope half-life and decay mode plus kinetic information of antibody uptake and tumour retention.

From the results it is self-evident that a high tumour uptake contributes to increased tumour dose. Less obvious is the dosimetric consequences of the interplay between isotope half-life, antibody uptake and subsequent loss from the tumour. A long-lived isotope gives a low body dose when en route to the target and allows more time for clearance from normal tissues while tumour irradiation is proceeding. Both these effects act to increase tumour specificity and depend upon a prolonged tumour residence time for the isotope. Currently the isotope which appears most suitable as a toxic radiolabel is the 64 h half-life beta emitter, 90-Yttrium, incorporated in a chelate link (as with 111-Indium) onto the antibody.

### Boron slow neutron capture with colloidal cobalt boride conjugated to McAb

C.H. Poynton, R.S. Tilbury, J. Head, S.E. Tindle, K.A. Dicke, L.J. Peters & C.L. Reading

*University of Texas MD Anderson Hospital and Tumor Institute, Houston, Texas, USA.*

In the course of preparing colloidal immunomagnetic fluids for cell separation in bone marrow transplantation we have found it possible to incorporate a large amount of boron into the colloidal particles in the form of cobalt boride. Cobalt chloride can be reduced by sodium borohydride to form colloidal cobalt boride particles of an even size (electron microscopy), which can be varied from 22–55 nm in diameter. The colloid is made in a human albumin solution which is then cross linked around the particles with glutaraldehyde followed by borohydride reduction. A McAb is then coupled to the albumin with benzoquinone. Emission spectroscopy of the lyophilized immunocolloid in 10% serum shows a B:Co ratio of 1:11 by weight. We have used the McAb CF-1 on the boride colloid, which reacts with close to 100% of K562 erythroleukaemic cells (grown in boron-free cell culture medium). The cells are thoroughly washed after the incubation with the colloid. By this method a boron content of 120 nMoles was obtained per $10^7$ cells lyophilized, giving a theoretical number of $1.4 \times 10^9$ $^{10}$B atoms per cell. We are in the process of measuring the boron content of cells incubated with non-reactive colloid and are now attempting to determine the flux of slow neutrons required to kill only colloid coated K562 cells: Viability is measured in a leukaemic cell colony assay since K562 cells have a high plating efficiency. Thermal neutron beams are generated at the TRIGA nuclear reactor at Texas A & M University with fluxes up to $1.7 \times 10^9$ n cm$^{-2}$ sec$^{-1}$. We have also measured $^{24}$Na production since the cells are suspended in PBS. We are investigating the toxicity of the procedure to normal haemopoietic progenitors with clonogenic assays. Further neutron irradiations are in progress.

### ABSTRACTS OF POSTER EXHIBITS

### Identification of bladder carcinoma cells by McAbs

R. Arndt, H. Huland, H. Dürkop, F. Donn & T. Löning

*Department of Immunology, Medical Clinic, Departments of Urology and Pathology, University of Hamburg, W Germany*

In order to establish McAbs for immunotherapy of bladder cancer we generated a series of hybridomas by fusion of B-cells from Balb/c mice immunized with human bladder carcinoma cell lines (Mano, 486 P). Here we present the data of two McAbs. Antibody Mano 4/4 (isotype IgG1) reacts with a 28 kd cell surface protein of bladder cancer cells. Immunohistology revealed a strong reactivity of Mano 4/4 in 18/19 bladder cancer. The antigen, designated UP 28, is predominantly expressed on those tumour cells which show invasive growth. UP 28 is expressed in low concentration on a subpopulation of normal bladder cells which is located in the basal layer of the epithelium. Antibody 486 P-3-12-1 reacts strongly with a cell surface antigen which is found on 17/19 bladder tumours so far tested. This antigen is expressed on single bladder epithelial cells which are located in foci in the intermediate and/or superficial layer of the normal bladder epithelium. The expression of the antigen on these cells is of particular interest, because of the focal appearance of bladder cancer. Higher density on the cell surface and expression on the vast majority of bladder cancers independent of grading and staging favours Mano 4/4 and 486 P-3-12-1 as candidates for immunotherapeutical application.

**Expression of the T67 antigen on B cell malignant disorders. Correlation with other immunological markers**

G. Laurent, E. Kuhlein[1], J.F. Brunet[1] & G. Delsol[2]

*Centre de Recherche Clin-Midy-SANOFI, Montpellier, [1]Laboratoire d'Immunologie, Toulouse, [2]CHU Purpan, Toulouse, France.*

Several McAbs (T101, leu 1, 10-2) have been described to detect 67,000–71,000 antigen (Tp67 molecule) present on normal and malignant T lymphocytes. The presence of this antigen on neoplastic B cells is also well established.

Nevertheless, little attention has been given to the relationship with other cell markers. Using immunofluorescence, 56 cases of B-CLL cells were investigated: the mean percentage value of Tp67 +B-CLL was 82.7%±15%. The mean value of $Tp67^+OKT3^-$ cells was 73%±18%, thus well correlated with the mean of surface Ig $(SIg)^+$ cells (77%±19%). No correlation between the percentage of $Tp67^+$ cells and serum Ig levels, monoclonal protein spike, SmIg phenotype, or clinical staging was found, whereas a slight correlation with mouse rosette forming cells was observed.

Using immunoperoxidase technique on frozen sections, 69 cases of B cell lymphoma were studied: Tp67 antigen was found in 24 cases, mostly in low grade lymphomas. In follicular lymphomas. two results deserve attention: (1) $T101^+$ lymphomas most frequently showed $IgM^+IgD^+SIg$. Inversely, T101 unreactive lymphomas displayed $IgM^+IgD$-phenotype. (2) Tp67 antigen and CALLA (Gp 100) were found to be mutually exclusive. These findings are important in the context of B cell differentiation pathways.

**Human placental alkaline phosphatase (hPLAP) as a tumour marker in serum and tissue extracts**

D.E. Pollett, E.J. Nouwen, J. Schelstraete, A. Van de Voorde[1], P. Blockx[2] & M.E. De Broe

*Department of Nephrology and [2]Department of Nuclear Medicine, University of Antwerp, and [1]Department of Molecular Biology, State University of Ghent, Belgium*

An enzyme antigen immunoassay based on a highly specific McAb against hPLAP was used to quantify hPLAP in: serum of blood donors, serum of unselected hospital patients, serum of cancer patients, butanol extracts of normal tissue, and butanol extracts of surgical tumour biopsies. Carcinoembryonic antigen (CEA) was determined by RIA when increased levels of hPLAP were found.

The normal reference limit ($P_{98}$ value) in serum of healthy blood donors (n = 117) was $0.05\,IU\,l^{-1}$, in serum of unselected hospital patients (n = 1650) it was $0.1\,IU\,l^{-1}$. In tissue extracts the $P_{98}$ value was $0.8\,mU\,g^{-1}$ tissue (lung tissue excluded). Normal lung tissue had a higher hPLAP content than all other normal tissues examined.

We found that 9.8% of all cancer patients, and 40% of ovarian cancer patients had increased hPLAP serum levels. Increased hPLAP in serum ($>0.1\,IU\,l^{-1}$) was accompanied by increased CEA ($>5\,ng\,ml^{-1}$) in 50% of all cases analysed (n = 20). Serum hPLAP levels of $>0.2\,IU\,l^{-1}$ were always an indication for an important tumour load. Increased hPLAP was found in 49% of all tumour biopsies and in 90% of ovarian neoplasia biopsies.

**First and second antibodies for the radioimmunodetection of tumours**

A.R. Bradwell, D.S. Fairweather, A. Keeling, P. Hawker, A. Vaughan & P.W. Dykes

*Department of Immunology, Medical School, Birmingham University, UK*

The major limiting factor in the detection of tumours using radiolabelled antibodies is the ratio of the antibody bound to the tumour compared with that in the surrounding normal tissues. Attempts to increase this ratio have centered on the use of affinity purified antibodies, McAbs and antibody fragments. An alternative approach is to reduce the non-bound antibody counts, particularly those present in the circulation. This has been achieved using a second antibody, both in a rat model and in humans. In the rat model 10 animals were given $^{131}$Iodine labelled sheep anti-CEA (A) and 24 h later pig anti-sheep IgG (B) or pig IgG (controls). After a further 24 h the circulating counts in the test rats had fallen to one-seventh of those in the controls. To understand the mechanism we incubated human macrophages with Igs from 5 different animals. Those Igs that bound to the Fc receptors (using a rosette test) on the macrophages, i.e. rabbit, pig and mouse, were considered useful second antibodies because immune complexes containing them would be rapidly catabolised. In contrast, antibodies with no uptake on human Fc receptors (sheep and goat) are useful primary antibodies because they do not falsely accumulate in macrophage containing tissues. We subsequently studied 3 patients with tumours. Two were given A and then a day later B (in a 15-fold molar excess). Over the next few hours the circulating counts fell 3 times faster than in patients not given second antibodies (the spleen accumulated the radioactivity) but tumour counts were maintained. The third patient was given a mouse McAb IgM anti-CEA first antibody and a sheep anti-mouse IgM second antibody. No increased clearance of circulating radioactivity occurred. Thus the use of a "clearing" second antibody led to improvements in tumour uptake ratios and to improved tumour detection.

**A comparison of radiolabelled McAbs and their $F(ab')_2$ fragments in direct radioimmunoassays and in xenografted nude mice**

H. Durbin, S.J. Mather, R.H. Raymond, E. Cliff & W.F. Bodmer

*Imperial Cancer Research Fund, Lincoln's Inn Fields, London, WC2A 3PX, UK*

$F(ab')_2$ fragments of two McAbs H17E2 and AUA-1 were prepared by pepsin digestion of the Ig and purified by affinity and size exclusion chromatography. Direct binding radioassays were performed on purified antigen preparations or relevant tumour cell lines either in solid phase or as live cell suspensions. Biodistribution experiments were performed in nude mice bearing colorectal tumour xenografts using radiolabelled Ig and $F(ab')_2$ preparations. Clearance rates, tumour uptake and tumour (T) to blood (B) and tumour to organ (O) ratios for the two antibody species were calculated. In general T:B and T:O ratios for $F(ab')_2$ were significantly higher than for intact antibody. $F(ab')_2$ clearance rates were faster while absolute tumour uptakes were similar.

These results suggest that, for diagnostic immunoscintigraphy, radiolabelled $F(ab')_2$ preparations are likely to be preferable to intact Igs.

**Antigen detection by McAbs following electromagnetic field exposures of human colon cancer cells**

W.D. Winters, M.P. Moyer & J.L. Phillips

*University of Texas Health Science Center and Cancer Therapy and Research Center, San Antonio, Texas 78284, USA*

Present information suggests that a major target site for electromagnetic (EM) field effects may be the cell membrane. We have used a set of McAbs produced to human colon cancer antigens as specific probes for changes in cells exposed to EM fields. Human colon cancer cells were exposed concurrently to EM fields ($E^+M^+$), magnetic fields alone ($M^+$), electric fields alone ($E^+$) and to no fields ($E^-M^-$) using a specially designed, standardized EM exposure facility ($E^+ = 300\,mA/M^2$ RMS; $M^+ = 1.0$ or 0.5 gauss RMS). Equal numbers of cells of each tumour type were exposed to the EM fields for 0, 6, 12 and 24 h at 37°C, resuspended in fresh medium and cultured for 3 days in 5% $CO_2$ at 37°C. Equal numbers of cells from each exposure group were reacted first with McAbs then with $^{125}I$-labelled anti-mouse IgG serum with appropriate wash cycles between. Radioactivity in final cell pellets was counted in a gamma counter.

Results showed that the level of antigens detected by McAbs markedly increased in colon cancer cells at the time of exposure to $E^+M^+$ and $M^+$ at 1.0 gauss increased, as compared to $E^-M^-$ controls. These data showed that time dependent exposures to $M^+$ and $E^+M^+$ fields induced marked changes in the levels of specific human colon cancer cell surface antigens.

Supported in part by New York State Department of Health Power Lines Project HRI-21082-07.

**Parameters influencing the uptake of radiolabelled McAb 96.5 in heterotransplanted malignant melanoma in nude rats**

K. Ydström, S.-E. Strand, C. Ingvar, P.-E. Jönsson, T. Brodin, H.-D. Sjögren & K. Wingardh

*Departments of Surgery, Radiation Physics and the Wallenberg Laboratory, University of Lund, Sweden*

In this study McAb 96.5, specific for melanoma antigen p97, was labelled with $^{125}I$ by three different techniques: Lactoperoxidase (LPO), Cloramine-T (CLT) and Bolton-Hunter (BH). As a control the antibody 1.4 (labelled with $^{131}I$) or human serum albumin was used for evaluation of non-specific uptake. The blood pool was evaluated in the tumour with *in vivo* $^{99}Tc^m$-labelled red blood cells. Blood flow measurements were performed with a microspheres technique.

Nude athymic rats, weighing 150–300 g (n = 70), transplanted with human melanoma were used as animal model. Scintillation camera measurements and blood samples were taken during 1 to 5 days. The rats were then sacrificed and tissue samples were measured for radioactivity. The specific activity uptake in the tumour for the McAb 96.5 was dependent on tumour wt. For tumours weighing <0.3 g the specific uptake was 2–3 times higher than for larger tumours. The biological half-life in the whole body was ~80–125 h for antibody 96.5 and 30–50 h for control antibody 1.4. Tumour/organ ratios were calculated and in the order of 0.3–0.6 for T/blood, 1.9–2.0 for T/liver and 3.3–4.6 for T/muscle.

This study was supported with grants from the Swedish Medical Research Council, Swedish Cancer Society and Ake Wibergs Foundation.

### Elimination of malignant cells from bone marrow in autologous rescue (ABMT)

R. Buckman, R.C. Coombes, A.J. Forester, V. Shepherd, R.A.J. McIlhinney & A.M. Neville

*Ludwig Institute of Cancer Research and Institute of Cancer Research, Royal Marsden Hospital, Sutton, Surrey, UK*

High dose chemotherapy is feasible when used with autologous bone marrow (BM) rescue. Malignant cells, however, that may be present in BM provide a potential source of future metastases. Immunological methods are being used as a BM "clean up" procedure.

We used an antibody (FIB-75) which recognises an antigen present on almost all epithelial tissues but not on BM stem cells. We demonstrated (*Lancet*, (1982) **ii**, 1428) that, in conjunction with exogenous rabbit complement (RC), this antibody can eliminate malignant cells in BM up to an infiltration of 1% with acceptable toxicity to CFU-C.

For large scale trials RC is technically awkward to use and thus we conjugated FIB to the A chains of ricin and abrin toxins. FIB-ricin at $10^{-7}$ M and FIB-abrin at $10^{-8}$ M showed abolition of protein synthesis and clonogenicity of all cell lines tested. There was acceptable toxicity to CFU-C (mean 62%, range 25–93%, N = 22) and mixed stem cell cultures (GEMM).

### Targeting of radiolabelled or toxin conjugated McAbs to human tumours

S. Canevari, S. Menard, S. Miotti, R. Orlandi, M. Ripamonti & M.I. Colnaghi

*Istituto Nazionale Tumori, Milan, Italy*

McAbs were raised against human ovary or breast cancer in the perspective of diagnostic and therapeutic clinical applications. After a complete study of their specificities we focused on two McAbs of IgM class, MRr1 and MOv2, respectively recognizing an antigen of epithelial cells of normal and cancerous mammary gland and an antigen present on epithelial ovarian carcinomas. *In vivo* distribution and half-life of McAbs in various conditions of injection and radiolabelling were studied in nude mice with transplanted mammary or ovarian tumour.

McAbs intravenously injected showed a very rapid elimination from the blood and no preferential uptake in subcutaneously growing tumours; McAbs intraperitoneally injected, in spite of a quite short half-life, localized in tumour nodules grown in the peritoneal cavity of pristane primed nude mice. The same McAbs have been developed as carriers of cytotoxic agents. Linkage of McAbs to ricin A chain has produced conjugates that display *in vitro* specific cytotoxicity for tumour cell lines with relevant target antigens. To plan a more efficient therapeutical administration of immunotoxins, kinetics of cytotoxicity have to be compared with *in vivo* McAbs' half-life.

### Induction of surface changes, membrane traffic alteration and growth delay on rat glioma cells by a McAb

T. Lederer, D. Stavrou[1], W. Mellert[1] & K.S. Zänker

*Institute of Experimental Surgery, Technical University, and [1]Department of Functional Pathology, University of Munich, W Germany*

In the investigation presented here, we examined the cell surface changes, the membrane traffic of 75-Se-methionine and growth delay of cultured rat glioma cells (79FR-G-41) in the presence of the McAb 14AC1. The production and characterization of the antibody used was described recently (Stavrou *et al.* (1983 *Eur. J. Cancer Clin. Oncol.*, **19**, 1439). Incubation of $5 \times 10^5$ cells with appropriate dilutions of the monoclonal antibody caused noticeable cell surface alterations. A major proportion of the Falcon flask attached cells, mostly growing flat, floated as spheres and lacked the commonly seen surface differentiations as microvilli and membrane ruffling; holes within the membrane were not observed by SCANS. Pre-incubation of the native cells with the antibody resulted in a diminished 75-Se-methionine uptake and the release of the label into the medium was dose-dependent reduced, when the antibody was allowed to react with cell surface structures. Only a small amount of the floating cells was dead. The remaining cells could be replated and attached to the flask, but did not continue to differentiate into their common morphological feature or to grow. These experiments suggest that the McAb alters cell surface structures, complement-independent, in such a way that detachment and growth delay occurs, likely due to arrested/suppressed membrane traffic of precursor molecules. Particular blocking during the cell cycle and/or shifting into $G_0$-phase is under current investigation.

**Cervical cancer: Differences in expression of cytokeratin polypeptides associated with malignant change**

C. A. Makin[1], L.G. Bobrow[1,2] & W.F. Bodmer[1]

[1]*Imperial Cancer Research Fund, London,* [2]*Department of Histopathology, University College Hospital, London, UK*

Antisera and McAbs recognising intermediate filaments are acknowledged to be of use in surgical pathology. Most workers, however, have utilised freshly frozen tissue sections or special fixatives. The McAb CAM5.2, which identifies the lower mol. wt. cytokeratin polypeptides found in secretory epithelia, has been shown to react consistently on formalin fixed paraffin embedded tissue sections. Preliminary studies using the immunoperoxidase technique indicated that CAM5.2 positively stained the endocervical glands of the normal cervix, as opposed to the ectocervix, which was negative: all invasive carcinomas of the cervix stained positively.

The change in expression of cytokeratin polypeptides with the development of malignancy in the cervix has been assessed in varying degrees of intraepithelial neoplasia and invasive carcinoma. Uniformly positive staining was noted in all invasive carcinomas, whereas the 10 cases of intraepithelial neoplasia studied were generally negative with some areas of equivocal staining. Our results suggest that as the extocervic becomes more dysplastic the cells start to manufacture the lower mol. wt., more embryonic forms of cytokeratin polypeptides. By the time the invasive stage is reached all cells are positive with CAM5.2 implying that the cells in the more malignant tumour have not differentiated in terms of their cytokeratin expression. CAM5.2 can be used clinically to distinguish normal ectocervical epithelium from invasive carcinoma and is of use in assessing the development of intra-epithelial neoplasia.

**Application of a McAb (H317) to detection of placental type alkaline phosphatase (PLAP) in ovarian cancer**

P.J. McLaughlin, M. Critchley, P.M. Tromans & P.M. Johnson

*Departments of Immunology, Nuclear Medicine and Obstetrics and Gynaecology, University of Liverpool, UK*

Placental-type alkaline phosphatases comprise a polymorphic group of enzymes in which two major forms can be distinguished: the "placental" and "placental-like" alkaline phosphatases (PLAP and placental-like AP, respectively). Both forms of the enzyme have been described as tumour products, and these correspond to the so-called Regan (PLAP) and Nagao (placental-like AP) enzymes. We have studied in depth a McAb H317 reactive with PLAP but not with placental-like AP or any non-placental tissue alkaline phosphatase isoenzymes. This McAb has formed the basis of a solid-phase enzyme immunoassay detecting $\geqq 0.1\,U\,l^{-1}$ PLAP (McLaughlin (1983) *et al., Clin Chim. Acta,* **130,** 199). In this assay 26/65 (40%) of ovarian cancer sera had detectable PLAP whereas PLAP was not found in any control sera. It appears that the circulating form of enzyme in healthy individuals is the placental-like AP and is thus unreactive with H317 (McLaughlin *et al.* (1984) *J. Clin. Pathol.,* in press). Thus, H317 may detect a more specific tumour marker within this enzyme group. The H317 McAb is also being used in *in vivo* antibody-guided radioimaging of ovarian cancer patients. The aim of this study is to evaluate $^{123}$I-labelled H317 McAb as a radioimaging agent for epithelial ovarian tumours 1–4 years after removal of the primary. The patient is injected i.v. with 74 MBq (2 mCi) $^{123}$I-H317 (2 mg mouse IgG). Using a large field of view gamma camera, anterior and posterior images are made at 10 min, 4 h and 24 h. In a preliminary study on 9 patients, three patterns of distribution were seen, *viz.* (i) focal accumulation of the $^{123}$I-H317 in areas of secondary involvement, (ii) diffuse uptake in abdomen and pelvis, and (iii) "cold" areas. Surgical confirmation of recurrence was possible in some cases.

### Heterogeneity in small cell lung carcinomas

F.M. Moss, L.G. Bobrow, M. Sheppard, D. Rowe, R.L. Souhami & P.C.L. Beverley

*ICRF Human Tumour Immunology Group, University College, London, UK*

Small cell carcinomas of the lung are a group of bronchial tumours distinct from non-small cell carcinomas both in their clinical behaviour and therapeutic response. Usually regarded as a single class there is, nevertheless, histological, clinical and therapeutic heterogeneity within the group. A panel of McAbs has been used to stain a series of small cell carcinomas. This has revealed heterogeneity both within and between tumours. Characterisation of antigenic heterogeneity may provide an additional method of classification of tumours within this group.